# Eight new species of the genus *Ischnothyreus* Simon, 1893 (Araneae, Oonopidae) from Hainan Island, China

**DOI:** 10.3897/zookeys.1286.201862

**Published:** 2026-07-24

**Authors:** Songlu Shi, Dongju Bian, Yanfeng Tong, Shuqiang Li

**Affiliations:** 1 College of Life Science, Shenyang Normal University, Shenyang 110034, Liaoning, China CAS Key Laboratory of Forest Ecology and Silviculture, Institute of Applied Ecology, Chinese Academy of Sciences Shenyang China; 2 CAS Key Laboratory of Forest Ecology and Silviculture, Institute of Applied Ecology, Chinese Academy of Sciences, Shenyang, 110016, China College of Life Science, Shenyang Normal University Shenyang China; 3 Forest Canopy Biodiversity Research Center, College of Life Sciences, Anhui Normal University, Wuhu, Anhui 241000, China Forest Canopy Biodiversity Research Center, College of Life Sciences, Anhui Normal University Wuhu China

**Keywords:** Goblin spiders, morphology, taxonomy

## Abstract

Eight new species of the genus *Ischnothyreus* Simon, 1893 are described from Hainan, China: *I. bawangling* Tong & Li, **sp. nov**. (♂♀), *I. diaoluoshan* Tong & Li, **sp. nov**. (♂♀), *I. hongxin* Tong & Li, **sp. nov**. (♂♀), *I. jianfengling* Tong & Li, **sp. nov**. (♂♀), *I. limuling* Tong & Li, **sp. nov**. (♂♀), *I. liudao* Tong & Li, **sp. nov**. (♂♀), *I. luobidong* Tong & Li, **sp. nov**. (♂♀) and *I. wuzhishan* Tong & Li, **sp. nov**. (♂). Diagnoses and illustrations for all eight species are given.

## Introduction

Oonopidae Simon, 1890, commonly known as goblin spiders, is a highly diverse spider family comprising 1990 extant described species in 115 genera. This family has a nearly global distribution, with the highest species richness in tropical and subtropical regions (WSC 2026). *Ischnothyreus* Simon, 1893 ranks among the most species-rich genera within Oonopidae, containing 129 extant species that are distributed mainly in East and South Asia, Australia, as well as three species that are recently introduced elsewhere (WSC 2026). Multiple taxonomic studies have contributed to our knowledge of *Ischnothyreus*, including descriptions of new species from Australia (Edward and Harvey 2014), China (e.g. Tong and Li 2012, 2014; Tong et al. 2018, 2021, 2023; Liu et al. 2019; Huang et al. 2021), and Southeast Asia (Kranz-Baltensperger 2011, 2012; Tong and Li 2013; Richard et al. 2016; Tong et al. 2016, 2020).

To date, 31 species of this genus have been documented from China (Fu et al. 2023; Song et al. 2024), 10 of which occur on Hainan Island (Tong and Li 2008, 2012). In the present study, we describe eight new species collected during recent field surveys.

### Materials and methods

The individuals were collected by sifting leaf litter. The specimens were examined using a Leica M205 C stereomicroscope. Details of body parts and measurements were studied under an Olympus BX51 compound microscope. Male and female internal genitalia were mounted on excavated slides with lactic acid and subsequently examined. Photographs were taken with a Canon EOS 750D zoom digital camera (18 megapixels) mounted on an Olympus BX51 compound microscope and assembled using Helicon Focus v. 3.10.3 image-stacking software (Khmelik et al. 2005). Scanning electron microscope images (SEM) were taken under high vacuum with a Hitachi S-4800 after critical-point drying and gold-palladium coating. All measurements in the text are expressed in millimetres. Type material is deposited in the Shenyang Normal University (**SYNU**) in Shenyang, Liaoning Province, China (curator: Yanfeng Tong).

Taxonomic descriptions follow Tong et al. (2021). The following abbreviations are used in the figures and text: **a** = apodeme; **ALE** = anterior lateral eyes; **bss** = bell-shaped structure; **css** = cone-shaped structure; **dhb** = disk-like hair base; **dm** = dorsal membrane; **gls** = goblet-like structure; **lhb** = large hair base; **PLE** = posterior lateral eyes; **PME** = posterior median eyes; **rl** = retrolateral lobe; **ssp** = slightly sclerotized process; **stp** = strong, tooth-like projection; **th** = thick hair; **tlp** = thorn-like process; **vl** = ventral lobe; **vp** = ventral projection; **vpr** = ventral protuberances; **wd** = winding duct.

## Taxonomy

### Family Oonopidae Simon, 1890

**Genus *Ischnothyreus* Simon, 1893**

#### Ischnothyreus bawangling

Taxon classificationAnimaliaAraneaeOonopidae

Tong & Li sp. nov.

6C917560-8FFC-5D2D-B993-C66BBFF0EA16

https://zoobank.org/9A4A891E-7751-4BC1-BD3D-BD44667A7DBE

**Figure 1.**
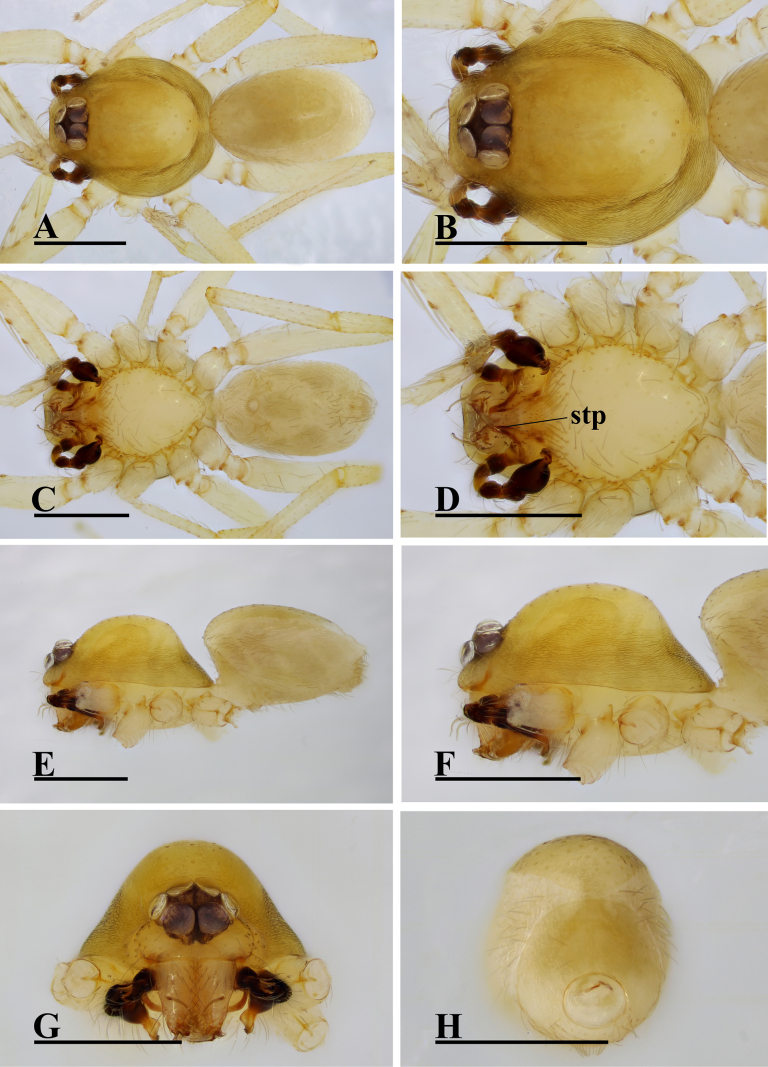
*Ischnothyreus bawangling* sp. nov., male holotype. **A**. Habitus, dorsal view; **B**. Cephalothorax, dorsal view; **C**. Habitus, ventral view; **D**. Cephalothorax, ventral view; **E**. Habitus, lateral view; **F**. Cephalothorax, lateral view; **G**. Cephalothorax, anterior view; **H**. Abdomen, anterior view. Abbreviation: stp = strong, tooth-like projection. Scale bars: 0.4 mm.

**Figure 2.**
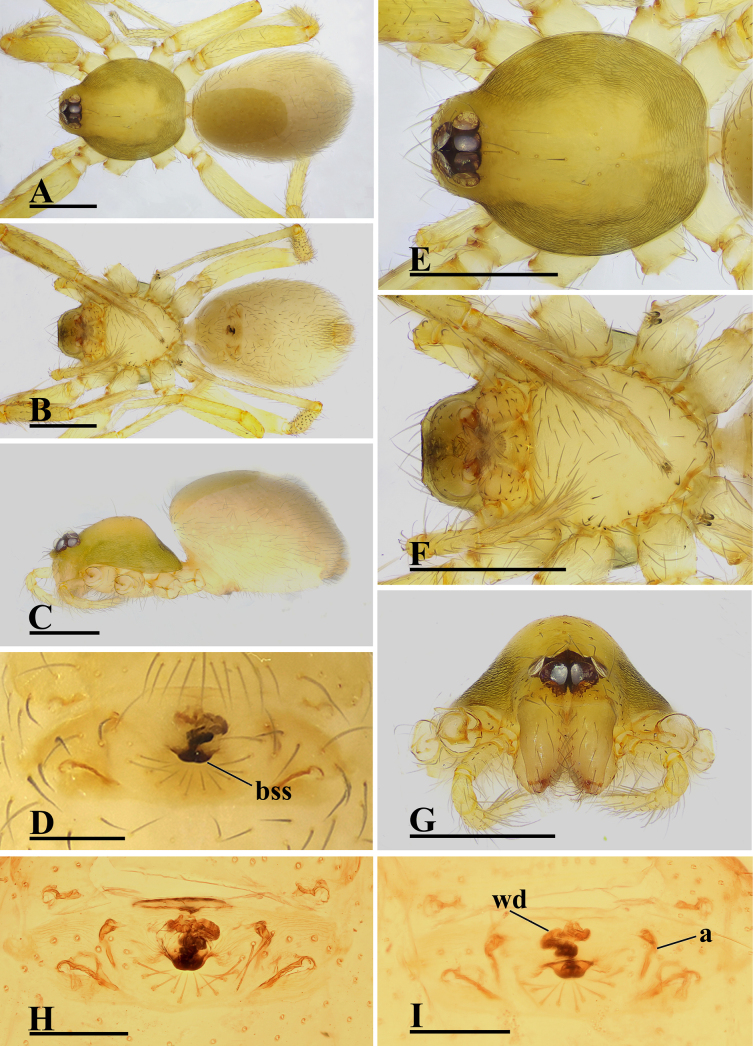
*Ischnothyreus bawangling* sp. nov., female paratype. **A**. Habitus, dorsal view; **B**. Habitus, ventral view; **C**. Habitus, lateral view; **D**. Epigastric region, ventral view; **E**. Cephalothorax, dorsal view; **F**. Cephalothorax, ventral view; **G**. Cephalothorax, anterior view; **H**. Endogyne, ventral view (cleared); **I**. Endogyne, dorsal view (cleared). Abbreviations: a = apodeme; bss = bell-shaped structure; wd = winding duct. Scale bars: 0.4 mm (**A–C, E–G**); 0.1 mm (**D, H, I**).

Figs 1, 2, 16 A, 17A, I, 18A–C, 20A–D

##### Common name.

Bawangling Weak-spotted Spider (霸王岭弱斑蛛).

##### Type material.

***Holotype***: China • ♂ (SYNU-F-1687); Hainan, Changjiang Li Autonomous County, Bawangling National Natural Reserve; 19°05'52.04"N, 109°10'39.94"E, 1007 ± 16 m elev.; 11.IV.2012; Chen leg. ***Paratypes***: China • 1♂ (SYNU-F-1686); same data as holotype • 1♂1♀ (SYNU-2000–2001); same data as holotype.

##### Etymology.

The specific name is a noun in apposition taken from the type locality.

##### Diagnosis.

The new species is similar to *I. falcatus* Tong & Li, 2008 in the morphology of the palpal bulb but can be distinguished by the smoothly curved thorn-like process on the male chelicerae (vs sickle-shaped; cf. Figs 16A, 17A, 17Iand Tong and Li 2008: fig. 2E), the dorsal scutum occupying approximately 4/5 of the abdomen length (vs 2/3; cf. Fig. 1A and Tong and Li 2008: fig. 2A), and the simple winding duct (vs complex; cf. Fig. 2I and Tong and Li 2008: fig. 2G).

##### Description.

**Male (holotype). *Body***: habitus as in Fig. 1A, C, E; body length 1.42. ***Carapace***: 0.73 long, 0.62 wide; yellow, broadly oval in dorsal view, with egg-shaped patches behind eyes, pars cephalica strongly elevated in lateral view, surface of elevated portion of pars cephalica smooth, sides finely reticulate (Fig. 1B, F). ***Clypeus***: straight in frontal view, ALE separated from edge of carapace by 0.8× of their diameter (Fig. 1G). ***Eyes***: six, well developed, ALE largest, ALE circular, PLE oval, PME squared; posterior eye row procurved from above; ALE touching, PME–PLE touching, ALE–PLE touching (Fig. 1B, G). ***Sternum***: heart-shaped, longer than wide, pale yellow, surface smooth, sparse setae (Fig. 1D). ***Mouthparts***: chelicerae, endites and labium yellow; chelicerae straight, anterior face with thorn-like process (tlp), base of fangs with slightly sclerotized process (ssp), fang groove with a few small and one larger denticles (Figs 1G, 16A, 17A, 17I); anteromedian tip of endites with one strong, tooth-like projection (stp) (Fig. 1D). ***Abdomen***: 0.74 long, 0.44 wide; dorsal scutum well sclerotized, yellow, covering 2/3 of abdomen width and approximately 4/5 of abdomen length; epigastric scutum, pale yellow, fused to postgastric scutum; postgastric scutum covering 2/3 of abdomen length (Fig. 1A, C, E). ***Legs***: pale yellow, leg spines longer than segment width, spine formula: femora: I p0-1-1; II p0-0-1; tibiae: I, II p2-1-1; r2-1-1; metatarsi: I, II p1-1-0; r1-1-0. Legs III and IV spineless. ***Palp***: dark reddish brown, trochanter with ventral projection (vp); bulb with two ventral protuberances (vpr), distal end of bulb stout, with large ventral lobe (vl), dorsal membrane (dm) and retrolateral lobe (rl) (Figs 18A–C, 20A–D).

**Female (paratype, SYNU-2001)**. Same as male except as noted. ***Body***: habitus as in Fig. 2A–C; body length 1.75. ***Carapace***: 0.76 long, 0.62 wide (Fig. 2E). ***Mouthparts***: chelicerae and endites unmodified (Fig. 2F, G). ***Abdomen***: 0.97 long, 0.66 wide; dorsal scutum covering ½ of abdomen width and approximately ½ of abdomen length; epigastric scutum well sclerotized, yellow, not fused to postgastric scutum. ***Endogyne***: with bell-shaped structure (bss); winding duct (wd) simple; apodemes (a) present (Fig. 2H, I).

##### Distribution.

Known only from the type locality.

#### Ischnothyreus diaoluoshan

Taxon classificationAnimaliaAraneaeOonopidae

Tong & Li sp. nov.

965D2A52-2D4A-5696-9EE5-97D01ABC4019

https://zoobank.org/7AA02EF0-F345-4BD6-A860-560839723180

**Figure 3.**
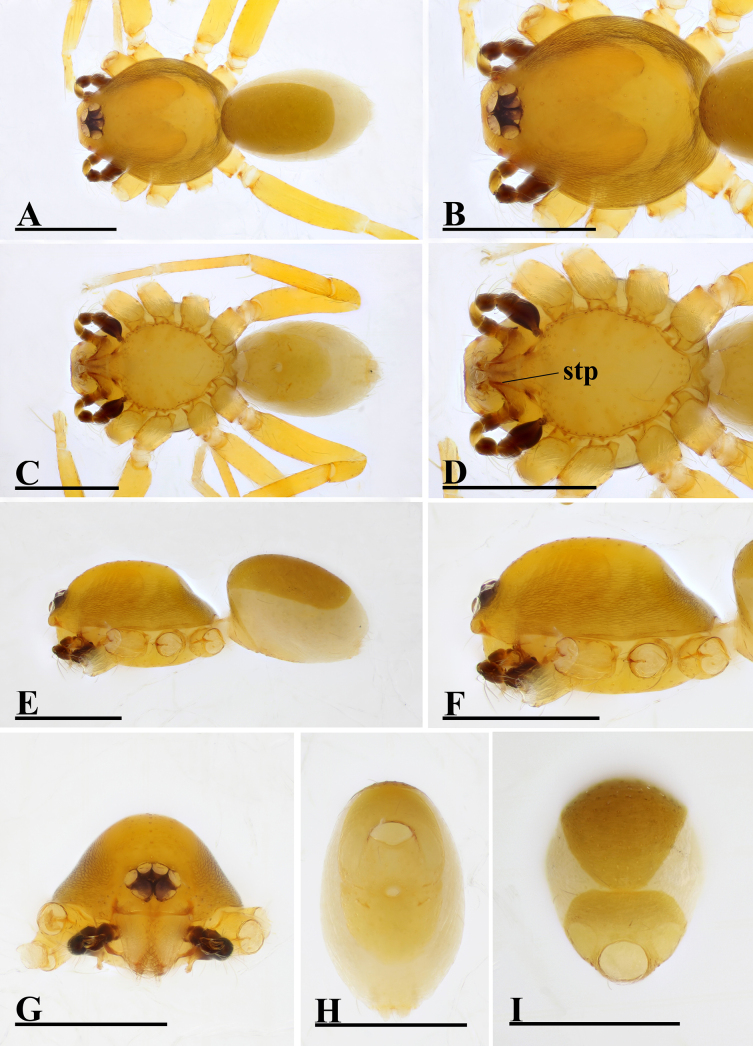
*Ischnothyreus diaoluoshan* sp. nov., male holotype. **A**. Habitus, dorsal view; **B**. Cephalothorax, dorsal view; **C**. Habitus, ventral view; **D**. Cephalothorax, ventral view; **E**. Habitus, lateral view; **F**. Cephalothorax, lateral view; **G**. Cephalothorax, anterior view; **H**. Abdomen, ventral view; **I**. Abdomen, anterior view. Abbreviation: stp = strong, tooth-like projection. Scale bars: 0.4 mm.

**Figure 4.**
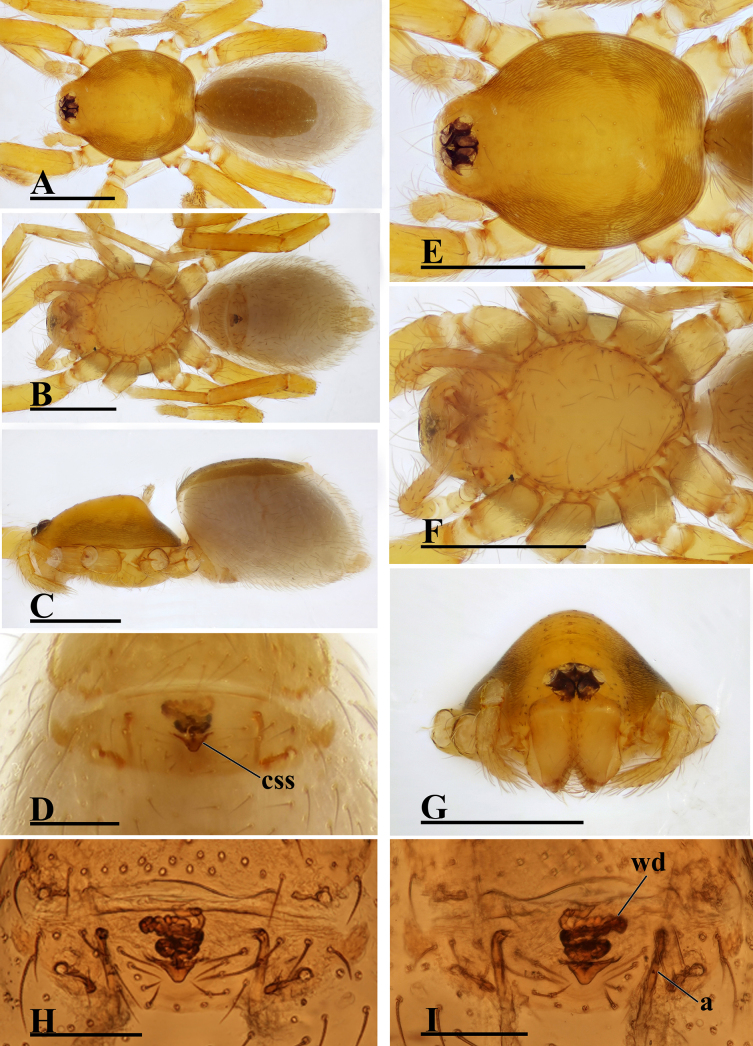
*Ischnothyreus diaoluoshan* sp. nov., female paratype. **A**. Habitus, dorsal view; **B**. Habitus, ventral view; **C**. Habitus, lateral view; **D**. Epigastric region, ventral view; **E**. Cephalothorax, dorsal view; **F**. Cephalothorax, ventral view; **G**. Cephalothorax, anterior view; **H**. Endogyne, ventral view (cleared); **I**. Endogyne, dorsal view (cleared). Abbreviations: a = apodeme; css = cone-shaped structure; wd = winding duct. Scale bars: 0.4 mm (**A–C, E–G**); 0.1 mm (**D, H, I**).

Figs 3, 4, 16 B, 17B, 18D–F, 20E–H

##### Common name.

Diaoluoshan Weak-spotted Spider (吊罗山弱斑蛛).

##### Type material.

***Holotype***: China • ♂ (SYNU-F-2457); Hainan, Lingshui Li Autonomous County, Benhao Town, Diaoluoshan National Natural Reserve; 18°43'33.28"N, 109°52'16.36"E, 937 ± 16 m elev.; 4.IV.2012; Chen leg. ***Paratypes***: China • 1♀ (SYNU-F-2458); same data as holotype • 2♀ (SYNU-F-2459–2460); same data as holotype • 1♂3♀ (SYNU-F-2464–2467); same data as holotype • 1♀ (SYNU-F-2461); 18°43'45.66"N, 109°51'52.92"E, 1000 ± 22 m elev.; 3.IV.2012; Tong leg; other data same as holotype • 1♀ (SYNU-F-2472); same data as above • 2♀ (SYNU-F-2462–2463); 18°43'39.83"N, 109°51'11.52"E, 1008 ± 23 m elev.; 5.IV.2012; other data same as holotype.

##### Etymology.

The specific name is a noun in apposition taken from the type locality.

##### Diagnosis.

The new species is similar to *I. jianfengling* sp. nov. and *I. kentingensis* Tong & Li, 2014 in the cone-shaped structure of endogyne but can be distinguished from *I. jianfengling* by the needle-shaped dorsal membrane of male palp (vs finger-shaped; cf. Fig. 20H and Fig. 21H) and the smaller cone-shaped structure, which occupies 1/5 of the distance between apodemes (vs 1/3; cf. Fig. 4D and Fig. 8D). It can be distinguished from *I. kentingensis* by the unmodified male chelicerae (vs with two strong, thorn-like processes; cf. Fig. 16B and Tong and Li 2014: fig. 1H) and the cone-shaped structure occupying 1/5 of the distance between apodemes (vs 3/4; cf. Fig. 4D and Tong and Li 2014: fig. 2G, J).

##### Description.

**Male (holotype). *Body***: habitus as in Fig. 3A, C, E; body length 1.19. ***Carapace***: 0.64 long, 0.51 wide; yellowish brown, oval in dorsal view, with egg-shaped patches behind eyes, pars cephalica strongly elevated in lateral view, surface of elevated portion of pars cephalica smooth, sides finely reticulate (Fig. 3B, F). ***Clypeus***: straight in frontal view, ALE separated from edge of carapace by 0.8× of their diameter (Fig. 3G). ***Eyes***: six, well developed, ALE largest, ALE circular, PLE oval, PME squared; posterior eye row procurved from above; ALE touching, PME–PLE touching, ALE–PLE touching (Fig. 3B, G). ***Sternum***: longer than wide, yellow, surface smooth, sparse setae (Fig. 3D). ***Mouthparts***: chelicerae, endites and labium yellow; chelicerae straight, base of fangs with slightly sclerotized process (ssp), fang groove with a few small denticles (Figs 16B, 17B); anteromedian tip of endites with one strong, tooth-like projection (stp) (Fig. 3D). ***Abdomen***: 0.62 long, 0.37 wide; dorsal scutum well sclerotized, yellowish brown, covering 4/5 of abdomen width and approximately 3/4 of abdomen length, fused to epigastric scutum (Fig. 3I); epigastric scutum, yellow, fused to postgastric scutum; postgastric scutum covering 2/3 of abdomen length (Fig. 3H). ***Legs***: yellow, leg spines longer than segment width, spine formula: femora: I p0-1-1; II p0-0-1; tibiae: I, II p2-1-1; r2-1-1; metatarsi: I, II p1-1-0; r1-1-0. Legs III and IV spineless. ***Palp***: dark reddish brown, trochanter with ventral projection; bulb with two ventral protuberances, distal end of bulb stout, with large ventral lobe (vl), needle-like dorsal membrane (dm) and retrolateral lobe (rl) (Figs 18D–F, 20E–H).

**Female (paratype, SYNU-F-2458)**. Same as male except as noted. ***Body***: habitus as in Fig. 4A–C; body length 1.51. ***Carapace***: 0.68 long, 0.53 wide (Fig. 4E). ***Mouthparts***: chelicerae and endites unmodified (Fig. 4F). ***Abdomen***: 0.85 long, 0.55 wide; dorsal scutum covering 1/2 of abdomen width and approximately 2/3 of abdomen length; epigastric scutum well sclerotized, yellowish brown, not fused to postgastric scutum (Fig. 4D). ***Endogyne***: with cone-shaped structure (css); winding duct (wd) strongly convoluted; apodemes (a) present (Fig. 4H, I).

##### Distribution.

Known only from the type locality.

#### Ischnothyreus hongxin

Taxon classificationAnimaliaAraneaeOonopidae

Tong & Li sp. nov.

579E37DF-E0BF-58AD-B308-61B9CC45EFF9

https://zoobank.org/127ED767-68A7-42E2-B9F9-5129BE904341

**Figure 5.**
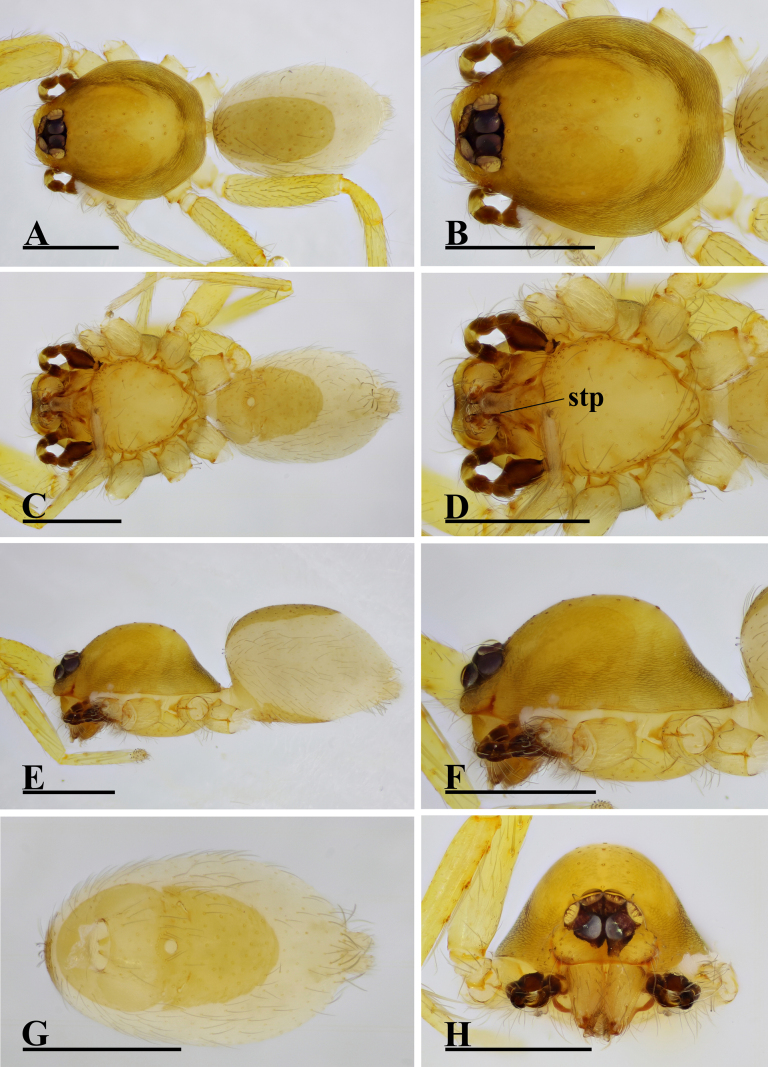
*Ischnothyreus hongxin* sp. nov., male holotype. **A**. Habitus, dorsal view; **B**. Cephalothorax, dorsal view; **C**. Habitus, ventral view; **D**. Cephalothorax, ventral view; **E**. Habitus, lateral view; **F**. Cephalothorax, lateral view; **G**. Abdomen, ventral view; **H**. Cephalothorax, anterior view. Abbreviation: stp = strong, tooth-like projection. Scale bars: 0.4 mm.

**Figure 6.**
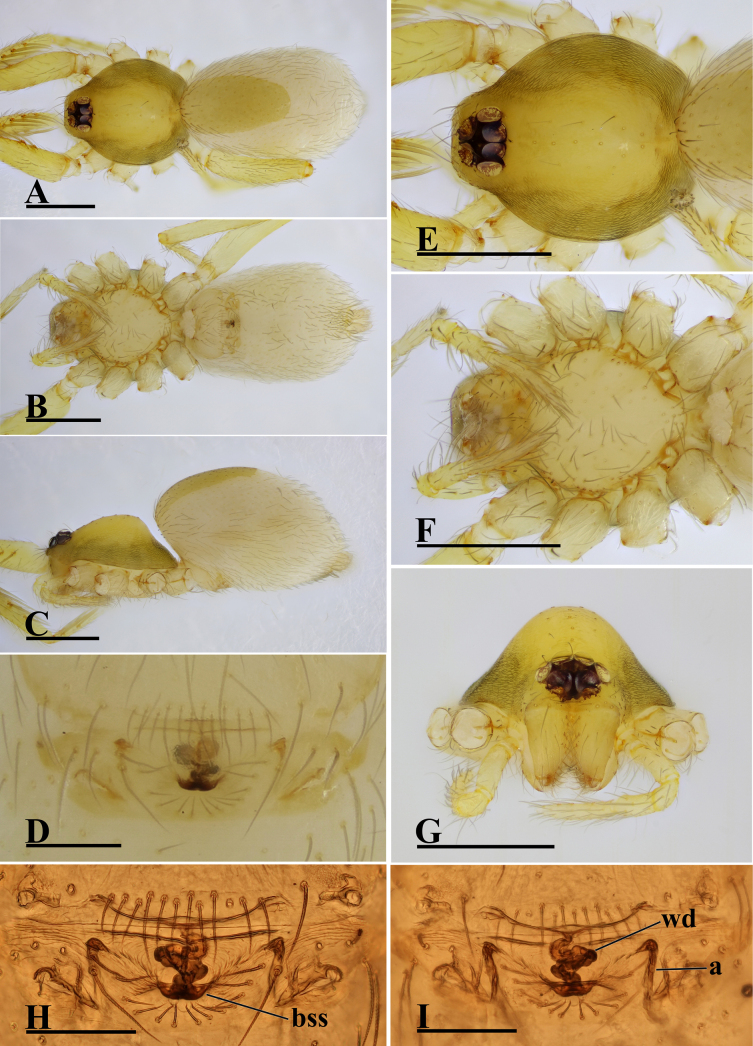
*Ischnothyreus hongxin* sp. nov., female paratype. **A**. Habitus, dorsal view; **B**. Habitus, ventral view; **C**. Habitus, lateral view; **D**. Epigastric region, ventral view; **E**. Cephalothorax, dorsal view; **F**. Cephalothorax, ventral view; **G**. Cephalothorax, anterior view; **H**. Endogyne, ventral view (cleared); **I**. Endogyne, dorsal view (cleared). Abbreviations: a = apodeme; bss = bell-shaped structure; wd = winding duct. Scale bars: 0.4 mm (**A–C, E–G**); 0.1 mm (**D, H, I**).

Figs 5, 6, 16 C, 17C, 18G–I, 21A–D

##### Common name.

Hongxin Weak-spotted Spider (红新弱斑蛛).

##### Type material.

***Holotype***: China • ♂ (SYNU-F-1519); Hainan, Baisha Li Autonomous County, Yuanmen Town, Yinggeling National Natural Reserve, Hongxin Vill.; 19°04'18.66"N, 109°31'45.12"E, 571 ± 23 m elev.; 27.III.2012; Chen leg. ***Paratypes***: China • 1♀ (SYNU-F-1520); same data as holotype • 1♀ (SYNU-F-1518); 19°04'15.24"N, 109°31'26.69"E, 511 ± 24 m elev.; 26.III.2012; other data same as holotype.

##### Etymology.

The specific name is a noun in apposition taken from the type locality.

##### Diagnosis.

The new species is similar to *I. campanaceus* Tong & Li, 2008 in the bell-shaped structure of endogyne but can be distinguished by the dorsal abdominal scutum covers approximately 2/3 of the abdomen length (vs nearly entirely abdominal length; cf. Figs 5A, 6Aand Tong and Li 2008: fig. 1A, B), the slightly sclerotized process of base of fangs (vs large, crown-like; cf. Figs 16C, 17Cand Tong and Li 2008: fig. 1D), and the ear-like dorsal membrane of palpal bulb (vs absent; cf. Figs 18G, 21Dand Tong and Li 2008: fig. 1G).

##### Description.

**Male (holotype). *Body***: habitus as in Fig. 5A, C, E; body length 1.52. ***Carapace***: 0.73 long, 0.59 wide; yellow, oval in dorsal view, with egg-shaped patches behind eyes, pars cephalica strongly elevated in lateral view, surface of elevated portion of pars cephalica smooth, sides finely reticulate (Fig. 5B, F). ***Clypeus***: straight in frontal view, ALE separated from edge of carapace by 1.0× of their diameter (Fig. 5H). ***Eyes***: six, well developed, ALE largest, ALE circular, PLE oval, PME squared; posterior eye row procurved from above; ALE touching, PME–PLE touching, ALE–PLE touching (Fig. 5B, H). ***Sternum***: heart-shaped, longer than wide, yellow, surface smooth, sparse setae (Fig. 5D). ***Mouthparts***: chelicerae, endites and labium yellow; chelicerae straight, anterior face with a thick hair (th), base of fangs with slightly sclerotized process (ssp), fang groove with a few small and one larger denticles (Figs 5H, 16C, 17C); anteromedian tip of endites with one strong, tooth-like projection (stp) (Fig. 5D). ***Abdomen***: 0.82 long, 0.49 wide; dorsal scutum well sclerotized, yellow, covering 1/2 of abdomen width and approximately 2/3 of abdomen length; epigastric scutum, yellow, fused to postgastric scutum; postgastric scutum covering 2/3 of abdomen length (Fig. 5G). ***Legs***: yellow, leg spines longer than segment width, spine formula: femora: I p0-1-1; II p0-0-1; tibiae: I, II p2-1-1; r2-1-1; metatarsi: I, II p1-1-0; r1-1-0. Legs III and IV spineless. ***Palp***: dark reddish brown, trochanter with ventral projection; bulb with two ventral protuberances, distal end of bulb stout, with leaf-like ventral lobe (vl), ear-like dorsal membrane (dm) and retrolateral lobe (rl) (Figs 18G–I, 21A–D).

**Female (paratype, SYNU-F-1520)**. Same as male except as noted. ***Body***: habitus as in Fig. 6A–C; body length 1.74. ***Carapace***: 0.74 long, 0.63 wide (Fig. 6E). ***Mouthparts***: chelicerae and endites unmodified (Fig. 6F). ***Abdomen***: 1.18 long, 0.66 wide; dorsal scutum covering 1/2 of abdomen width and approximately 1/2 of abdomen length; epigastric scutum well sclerotized, yellow, not fused to postgastric scutum (Fig. 6D). ***Endogyne***: with bell-shaped structure (bss); winding duct (wd) simple; apodemes (a) present (Fig. 6H, I).

##### Distribution.

Known only from the type locality.

#### Ischnothyreus jianfengling

Taxon classificationAnimaliaAraneaeOonopidae

Tong & Li sp. nov.

2CCC7A41-AC71-5646-9D24-CC4C18A98D96

https://zoobank.org/73CB1E95-5F9C-47B8-9ED5-2D15C9CE32CF

**Figure 7.**
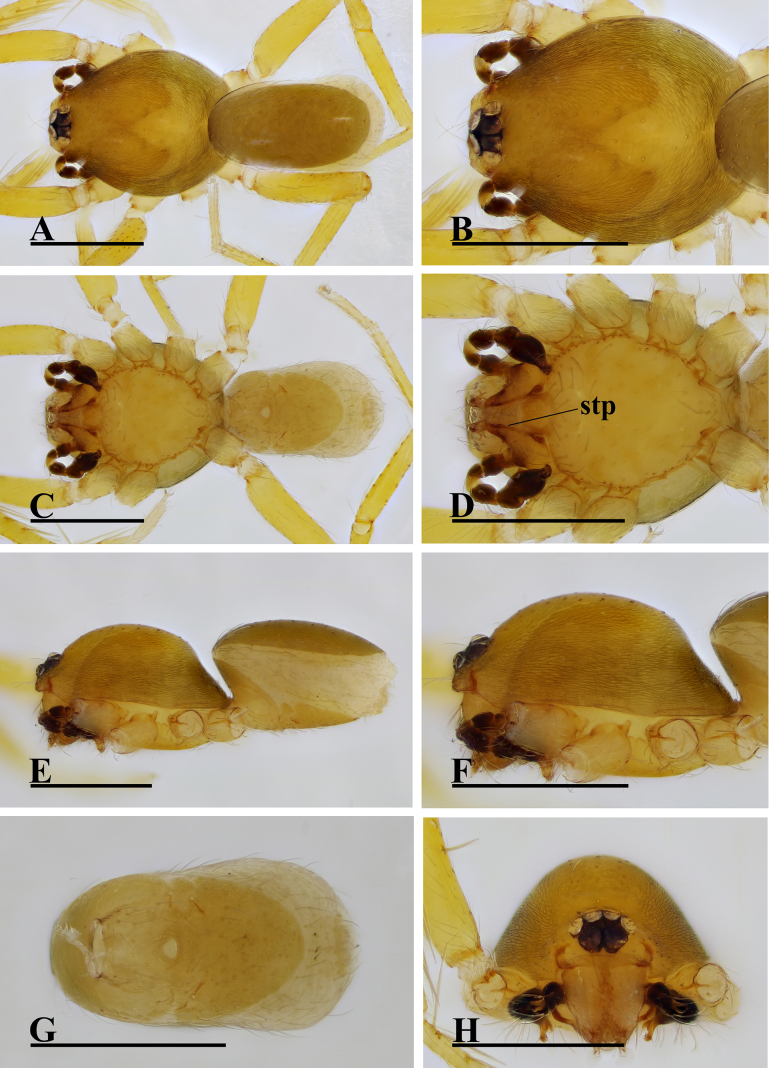
*Ischnothyreus jianfengling* sp. nov., male holotype. **A**. Habitus, dorsal view; **B**. Cephalothorax, dorsal view; **C**. Habitus, ventral view; **D**. Cephalothorax, ventral view; **E**. Habitus, lateral view; **F**. Cephalothorax, lateral view; **G**. Abdomen, ventral view; **H**. Cephalothorax, anterior view. Abbreviation: stp = strong, tooth-like projection. Scale bars: 0.4 mm.

**Figure 8.**
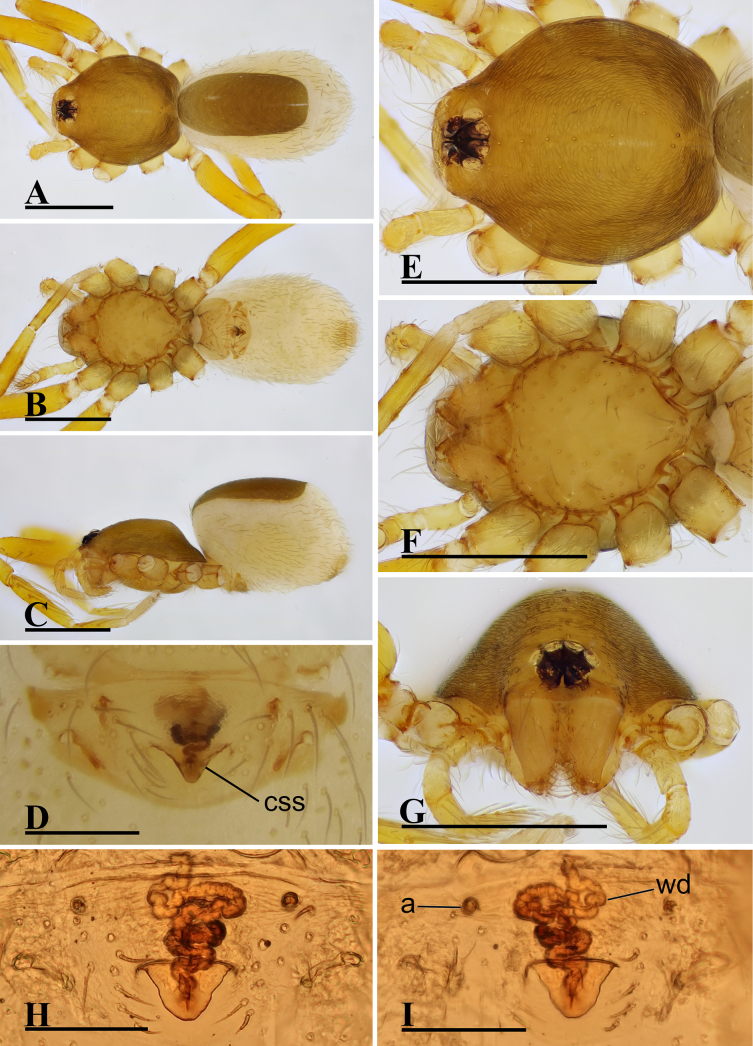
*Ischnothyreus jianfengling* sp. nov., female paratype. **A**. Habitus, dorsal view; **B**. Habitus, ventral view; **C**. Habitus, lateral view; **D**. Epigastric region, ventral view; **E**. Cephalothorax, dorsal view; **F**. Cephalothorax, ventral view; **G**. Cephalothorax, anterior view; **H**. Endogyne, ventral view (cleared); **I**. Endogyne, dorsal view (cleared). Abbreviations: a = apodeme; css = cone-shaped structure; wd = winding duct. Scale bars: 0.4 mm (**A–C, E–G**); 0.1 mm (**D, H, I**).

Figs 7, 8, 16 D, 17D, 18J–L, 21E–H

##### Common name.

Jianfengling Weak-spotted Spider (尖峰岭弱斑蛛).

##### Type material.

***Holotype***: China • ♂ (SYNU-1997); Hainan, Ledong Li Autonomous County, Jianfeng Town, Jianfengling National Forest Park, Tianchi Lake; 18°44'19.68"N, 109°52'01.02"E, 871 ± 18 m elev.; 7.IV.2012; Chen leg. ***Paratypes***: China • 2♀ (SYNU-1998–1999); same data as holotype • 3♂6♀ (SYNU-F-2434–2442); same data as holotype • 1♂3♀ (SYNU-F-2446–2450); same data as holotype • 1♀ (SYNU-F-2454); same data as holotype • 1♀ (SYNU-F-2455); same data as holotype • 2♂ (SYNU-F-2444–2445); 18°44'31.31"N, 109°52'07.82"E, 816 ± 40 m elev.; 6.IV.2012; other data same as holotype • 2♀ (SYNU-F-2452–2453); same data as above.

##### Etymology.

The specific name is a noun in apposition taken from the type locality.

##### Diagnosis.

The new species is similar to *I. diaoluoshan* sp. nov. and *I. kentingensis* Tong & Li, 2014 in the cone-shaped structure of endogyne but can be distinguished from *I. diaoluoshan* by the finger-shaped dorsal membrane of male palp (vs needle-shaped; cf. Fig. 21H and Fig. 20H) and the cone-shaped structure, which occupies 1/3 of the distance between apodemes (vs 1/5; cf. Fig. 8D and Fig. 4D). It can be distinguished from *I. kentingensis* by the unmodified male chelicerae (vs with two strong, thorn-like processes; cf. Fig. 16D and Tong and Li 2014: fig. 1H) and the cone-shaped structure occupying 1/3 of the distance between apodemes (vs 3/4; cf. Fig. 8D and Tong and Li 2014: fig. 2G, J).

##### Description.

**Male (holotype). *Body***: habitus as in Fig. 7A, C, E; body length 1.19. ***Carapace***: 0.65 long, 0.53 wide; brown, oval in dorsal view, with egg-shaped patches behind eyes, pars cephalica strongly elevated in lateral view, surface of elevated portion of pars cephalica smooth, sides strongly reticulate (Fig. 7B, F). ***Clypeus***: straight in frontal view, ALE separated from edge of carapace by 0.7× of their diameter (Fig. 7H). ***Eyes***: ALE largest, ALE circular, PLE oval, PME squared; posterior eye row straight from above; ALE touching, PME–PLE touching, ALE–PLE touching (Fig. 7B, H). ***Sternum***: longer than wide, yellow, surface smooth, sparse setae (Fig. 7D). ***Mouthparts***: chelicerae, endites and labium yellow; chelicerae straight, base of fangs with slightly sclerotized process (ssp), fang groove with a few small and one larger denticles (Figs 16D, 17D); anteromedian tip of endites with one strong, tooth-like projection (stp) (Fig. 7D). ***Abdomen***: 0.62 long, 0.35 wide; dorsal scutum well sclerotized, yellowish brown, covering almost all of abdomen width and approximately 7/8 of abdomen length; epigastric scutum, yellow, fused to postgastric scutum; postgastric scutum covering 5/6 of abdomen length (Fig. 7A, C, E, G). ***Legs***: yellow, leg spines longer than segment width, spine formula: femora: I p0-1-1; II p0-0-1; tibiae: I, II p2-1-1; r2-1-1; metatarsi: I, II p1-1-0; r1-1-0. Legs III and IV spineless. ***Palp***: dark reddish brown, trochanter with ventral projection; bulb with two ventral protuberances, distal end of bulb stout, with ventral lobe (vl), finger-like dorsal membrane (dm) and retrolateral lobe (rl) (Figs 18J–L, 21E–H).

**Female (paratype, SYNU-1998)**. Same as male except as noted. ***Body***: habitus as in Fig. 8A–C; body length 1.38. ***Carapace***: 0.61 long, 0.52 wide (Fig. 8E). ***Mouthparts***: chelicerae and endites unmodified (Fig. 8F). ***Abdomen***: 0.85 long, 0.51 wide; dorsal scutum covering 1/2 of abdomen width and approximately 3/4 of abdomen length; epigastric scutum well sclerotized, yellow, not fused to postgastric scutum (Fig. 8D). ***Endogyne***: with cone-shaped structure (css); winding duct (wd) strongly convoluted; apodemes (a) present (Fig. 8H, I).

##### Distribution.

Known only from the type locality.

#### Ischnothyreus limuling

Taxon classificationAnimaliaAraneaeOonopidae

Tong & Li sp. nov.

4188250B-FA70-5BAD-86F1-1523D2808B53

https://zoobank.org/98DF0ACA-74C8-4515-AD44-86346A0A32E5

**Figure 9.**
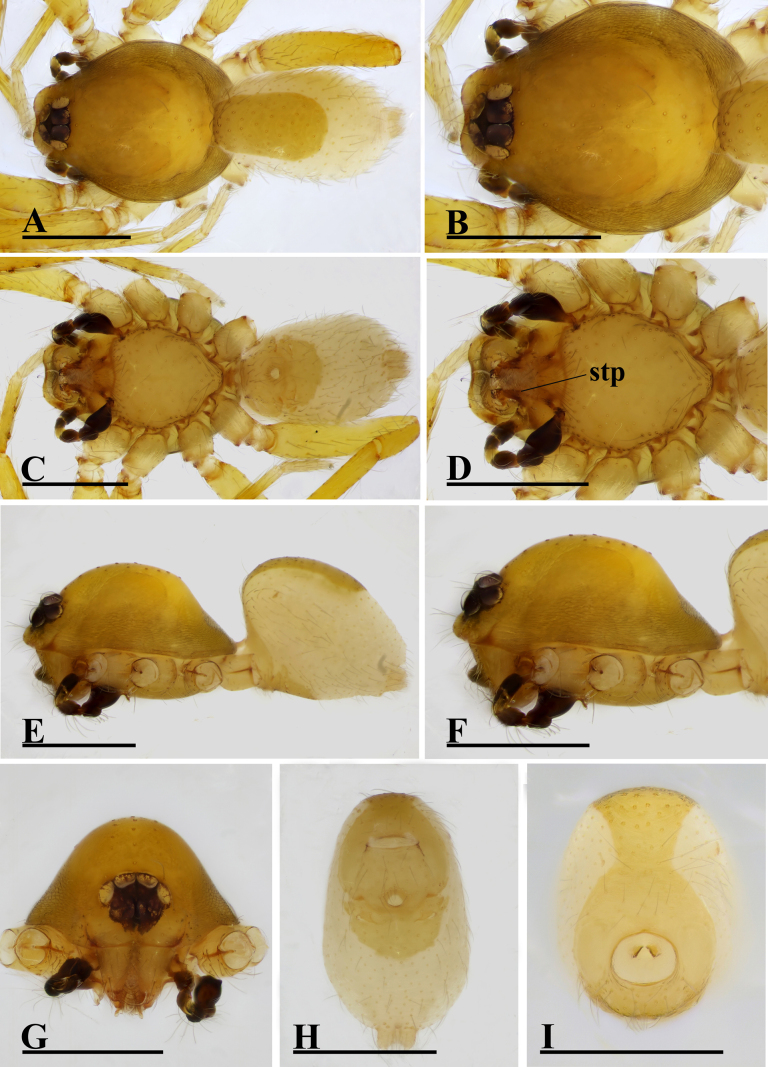
*Ischnothyreus limuling* sp. nov., male holotype. **A**. Habitus, dorsal view; **B**. Cephalothorax, dorsal view; **C**. Habitus, ventral view; **D**. Cephalothorax, ventral view; **E**. Habitus, lateral view; **F**. Cephalothorax, lateral view; **G**. Cephalothorax, anterior view; **H**. Abdomen, ventral view; **I**. Abdomen, anterior view. Abbreviation: stp = strong, tooth-like projection. Scale bars: 0.4 mm.

**Figure 10.**
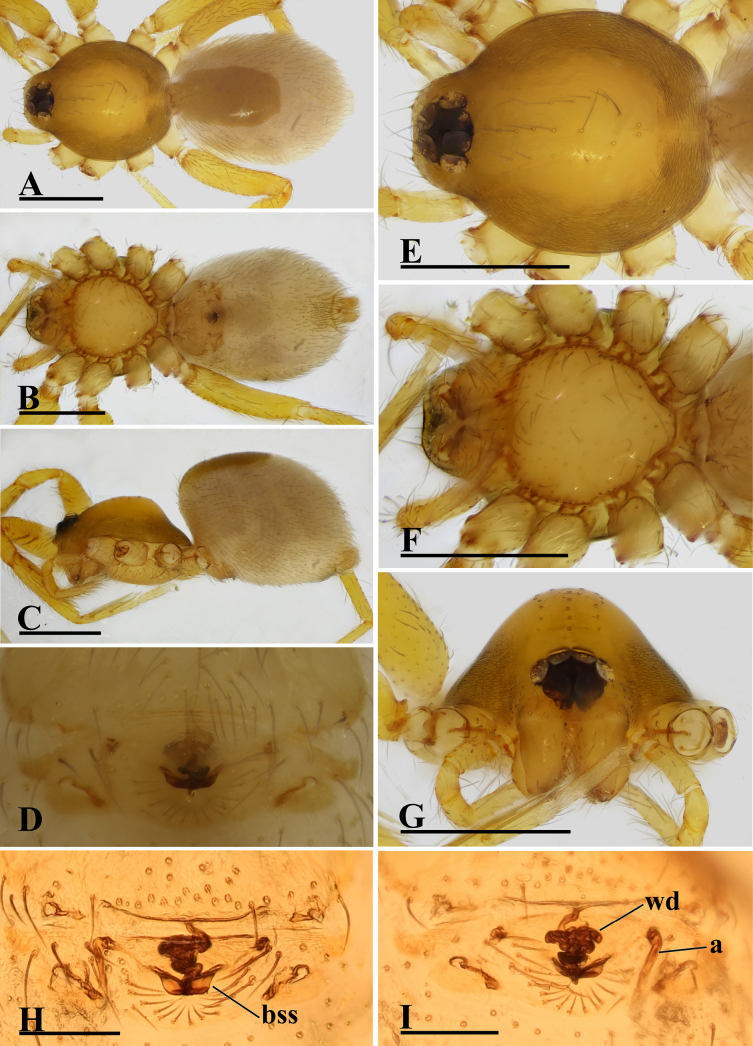
*Ischnothyreus limuling* sp. nov., female paratype. **A**. Habitus, dorsal view; **B**. Habitus, ventral view; **C**. Habitus, lateral view; **D**. Epigastric region, ventral view; **E**. Cephalothorax, dorsal view; **F**. Cephalothorax, ventral view; **G**. Cephalothorax, anterior view; **H**. Endogyne, ventral view (cleared); **I**. Endogyne, dorsal view (cleared). Abbreviations: a = apodeme; bss = bell-shaped structure; wd = winding duct. Scale bars: 0.4 mm (**A–C, E–G**); 0.1 mm (**D, H, I**).

Figs 9, 10, 16 E, 17E, 19A–C, 22A–D

##### Common name.

Limuling Weak-spotted Spider (黎母岭弱斑蛛).

##### Type material.

***Holotype***: China • ♂ (SYNU-F-2531); Hainan, Qiongzhong Li and Miao Autonomous County, Limushan Town, Limushan National Natural Reserve, Yinhe Protection Station; 19°12'00.11"N, 109°43'42.60"E, 591 ± 7 m elev.; 25.III.2012; Chen leg. ***Paratypes***: China • 1♂ (SYNU-F-2523); same data as holotype • 1♀ (SYNU-F-2532); same data as holotype • 3♀ (SYNU-F-2528–2530); 19°12'24.73"N, 109°44'12.41"E, 596 ± 10 m elev.; 24.III.2012; other data same as holotype.

##### Etymology.

The specific name is a noun in apposition taken from the type locality.

##### Diagnosis.

The new species is similar to *I. campanaceus* Tong & Li, 2008 in the bell-shaped structure of endogyne but can be distinguished by the dorsal abdominal scutum, which covers approximately 1/2 of abdomen length (vs nearly entirely abdominal length; cf. Figs 9A, 10Aand Tong and Li 2008: fig. 1A, B) and the dorsal abdominal scutum of male fused to the epigastric scutum (vs unfused; cf. Fig. 9I and Tong and Li 2008: fig. 1A).

##### Description.

**Male (holotype). *Body***: habitus as in Fig. 9A, C, E; body length 1.38. ***Carapace***: 0.76 long, 0.61 wide; brown, oval in dorsal view, with egg-shaped patches behind eyes, pars cephalica strongly elevated in lateral view, surface of elevated portion of pars cephalica smooth, sides finely reticulate (Fig. 9B, F). ***Clypeus***: straight in frontal view, ALE separated from edge of carapace by 1.1× of their diameter (Fig. 9G). ***Eyes***: six, well developed, ALE largest, ALE circular, PLE oval, PME squared; posterior eye row procurved from above; ALE touching, PME–PLE touching, ALE–PLE touching (Fig. 9B, G). ***Sternum***: heart-shaped, as long as wide, yellowish brown, surface smooth, sparse setae (Fig. 9D). ***Mouthparts***: chelicerae, endites and labium yellowish brown; chelicerae straight, base of fangs with slightly sclerotized process (ssp), fang groove with a few small and one larger denticles (Figs 16E, 17E); anteromedian tip of endites with one strong, tooth-like projection (stp) (Fig. 9D). ***Abdomen***: 0.73 long, 0.41 wide; dorsal scutum well sclerotized, yellowish brown, covering 1/2 of abdomen width and approximately 1/2 of abdomen length, fused to epigastric scutum (Fig. 9A, I); epigastric scutum, yellow, fused to postgastric scutum; postgastric scutum covering 3/5 of abdomen length (Fig. 9H). ***Legs***: yellow, leg spines longer than segment width, spine formula: femora: I p0-1-1; II p0-0-1; tibiae: I, II p2-1-1; r2-1-1; metatarsi: I, II p1-1-0; r1-1-0. Legs III and IV spineless. ***Palp***: trochanter with ventral projection; bulb with two ventral protuberances, distal end of bulb stout, with leaf-like ventral lobe (vl), ear-like dorsal membrane (dm) and retrolateral lobe (rl) (Figs 19A–C, 22A–D).

**Female (paratype, SYNU-F-2532)**. Same as male except as noted. ***Body***: habitus as in Fig. 10A–C; body length 1.60. ***Carapace***: 0.68 long, 0.59 wide (Fig. 10E). ***Mouthparts***: chelicerae and endites unmodified (Fig. 10F). ***Abdomen***: 0.98 long, 0.65 wide; dorsal scutum covering 1/3 of abdomen width and approximately 1/2 of abdomen length; epigastric scutum well sclerotized, yellowish brown, not fused to postgastric scutum (Fig. 10D). ***Endogyne***: with bell-shaped structure (bss); winding duct (wd) strongly convoluted; apodemes (a) present (Fig. 10H, I).

##### Distribution.

Known only from the type locality.

#### Ischnothyreus liudao

Taxon classificationAnimaliaAraneaeOonopidae

Tong & Li sp. nov.

ECEE72CB-CECC-53A9-A153-71F8D1915534

https://zoobank.org/9EF8808F-1B7E-41D5-A5DE-629815E0BAF4

**Figure 11.**
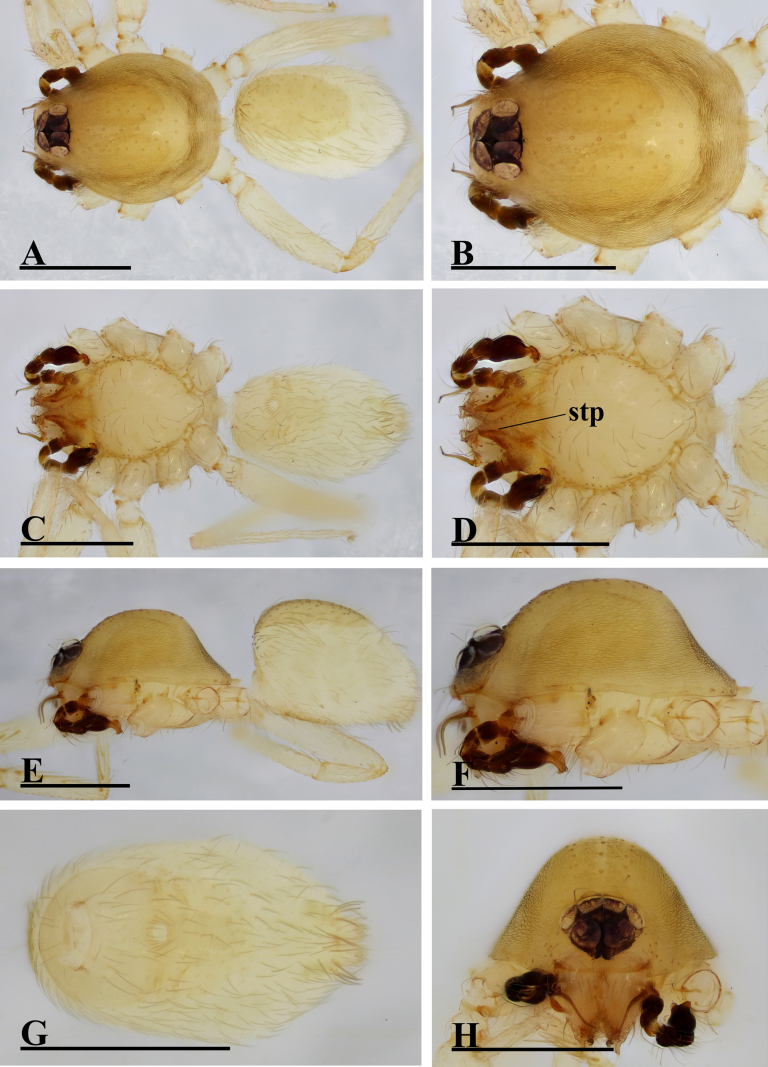
*Ischnothyreus liudao* sp. nov., male holotype. **A**. Habitus, dorsal view; **B**. Cephalothorax, dorsal view; **C**. Habitus, ventral view; **D**. Cephalothorax, ventral view; **E**. Habitus, lateral view; **F**. Cephalothorax, lateral view; **G**. Abdomen, ventral view; **H**. Cephalothorax, anterior view. Abbreviation: stp = strong, tooth-like projection. Scale bars: 0.4 mm.

**Figure 12.**
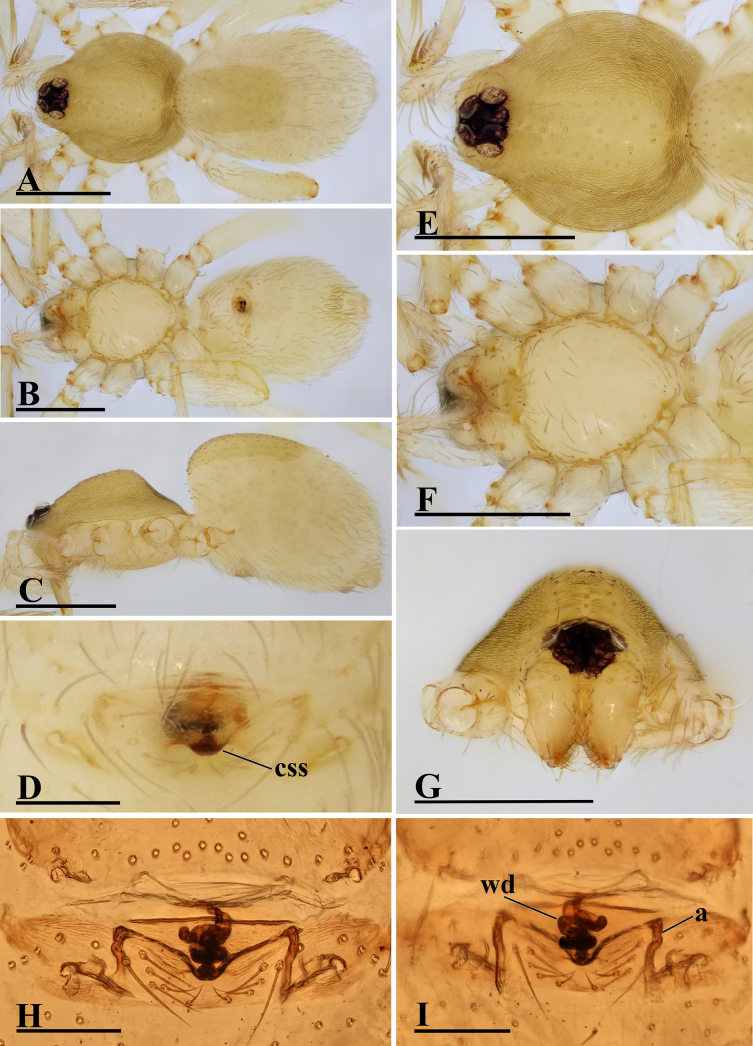
*Ischnothyreus liudao* sp. nov., female paratype. **A**. Habitus, dorsal view; **B**. Habitus, ventral view; **C**. Habitus, lateral view; **D**. Epigastric region, ventral view; **E**. Cephalothorax, dorsal view; **F**. Cephalothorax, ventral view; **G**. Cephalothorax, anterior view; **H**. Endogyne, ventral view (cleared); **I**. Endogyne, dorsal view (cleared). Abbreviations: a = apodeme; css = cone-shaped structure; wd = winding duct. Scale bars: 0.4 mm (**A–C, E–G**); 0.1 mm (**D, H, I**).

Figs 11, 12, 16 F, 17F, J, 19D–F, 22E–H

##### Common name.

Liudao Weak-spotted Spider (六道弱斑蛛).

##### Type material.

***Holotype***: China • ♂ (SYNU-F-2490); Hainan, Sanya City, Jiyang Dist., Liudao Vill., hill near the seaside; 18°12'34.50"N, 109°33'44.70"E, 17 ± 11 m elev.; 16.IV.2012; Tong leg. ***Paratypes***: China • 2♀ (SYNU-F-2491–2492): same data as holotype • 9♂2♀ (SYNU-2493–2503): same data as holotype.

##### Etymology.

The specific name is a noun in apposition taken from the type locality.

##### Diagnosis.

The new species is similar to *I. spineus* Tong & Li, 2012 in the thorn-like process of male chelicerae but can be distinguished by the dorsal abdominal scutum covers approximately 2/3 of the abdomen length (vs 4/5; cf. Figs 11A, 12Aand Tong and Li 2012: figs 3A, 4A), the palpal bulb has a large ventral lobe (vs absent; cf. Figs 19D, 22G, 22Hand Tong and Li 2012: figs 3I, 5B), and the endogyne possesses a cone-shaped structure (vs lacking, instead having a semicircular depression; cf. Fig. 12D and Tong and Li 2012: fig. 4G).

##### Description.

**Male (holotype). *Body***: habitus as in Fig. 11A, C, E; body length 1.36. ***Carapace***: 0.67 long, 0.55 wide; light yellow, oval in dorsal view, with egg-shaped patches behind eyes, pars cephalica strongly elevated in lateral view, surface of elevated portion of pars cephalica smooth, sides finely reticulate (Fig. 11B, F). ***Clypeus***: straight in frontal view, ALE separated from edge of carapace by 0.8× of their diameter (Fig. 11H). ***Eyes***: six, well developed, ALE largest, ALE circular, PLE oval, PME squared; posterior eye row procurved from above; ALE touching, PME–PLE touching, ALE–PLE touching (Fig. 11B, H). ***Sternum***: heart-shaped, as long as wide, pale yellow, surface smooth, sparse setae (Fig. 11D). ***Mouthparts***: chelicerae, endites and labium yellow; chelicerae straight, anterior face with thorn-like process (tlp) on large hair base (lhb), base of fangs with slightly sclerotized process (ssp), fang groove with a few small denticles (Figs 16F, 17F); anteromedian tip of endites with one strong, tooth-like projection (stp) (Fig. 11D). ***Abdomen***: 0.63 long, 0.40 wide; dorsal scutum well sclerotized, pale yellow, covering 1/2 of abdomen width and approximately 2/3 of abdomen length; epigastric scutum, pale yellow, fused to postgastric scutum; postgastric scutum covering 3/5 of abdomen length (Fig. 11G). ***Legs***: pale yellow, leg spines longer than segment width, spine formula: femora: I p0-1-1; II p0-0-1; tibiae: I, II p2-1-1; r2-1-1; metatarsi: I, II p1-1-0; r1-1-0. Legs III and IV spineless.***Palp***: dark reddish brown, trochanter with ventral projection; bulb with two ventral protuberances, distal end of bulb stout, with large ventral lobe (vl), dorsal membrane (dm) and retrolateral lobe (rl) (Figs 19D–F, 22E–H).

**Female (paratype, SYNU-F-2492)**. Same as male except as noted. ***Body***: habitus as in Fig. 12A–C; body length 1.45. ***Carapace***: 0.66 long, 0.56 wide (Fig. 12E). ***Mouthparts***: chelicerae and endites unmodified (Fig. 12F). ***Abdomen***: 0.86 long, 0.59 wide; dorsal scutum covering 1/2 of abdomen width and approximately 1/2 of abdomen length; epigastric scutum well sclerotized, pale yellow, not fused to postgastric scutum (Fig. 12A–D). ***Endogyne***: with cone-shaped structure (css); winding duct (wd) strongly convoluted; apodemes (a) present (Fig. 12H, I).

##### Distribution.

Known only from the type locality.

#### Ischnothyreus luobidong

Taxon classificationAnimaliaAraneaeOonopidae

Tong & Li sp. nov.

55637937-3EBC-5BB9-8E30-D8BBDCE0536C

https://zoobank.org/F92CA5B4-DCED-4A97-8FB3-89140F9E6F5F

**Figure 13.**
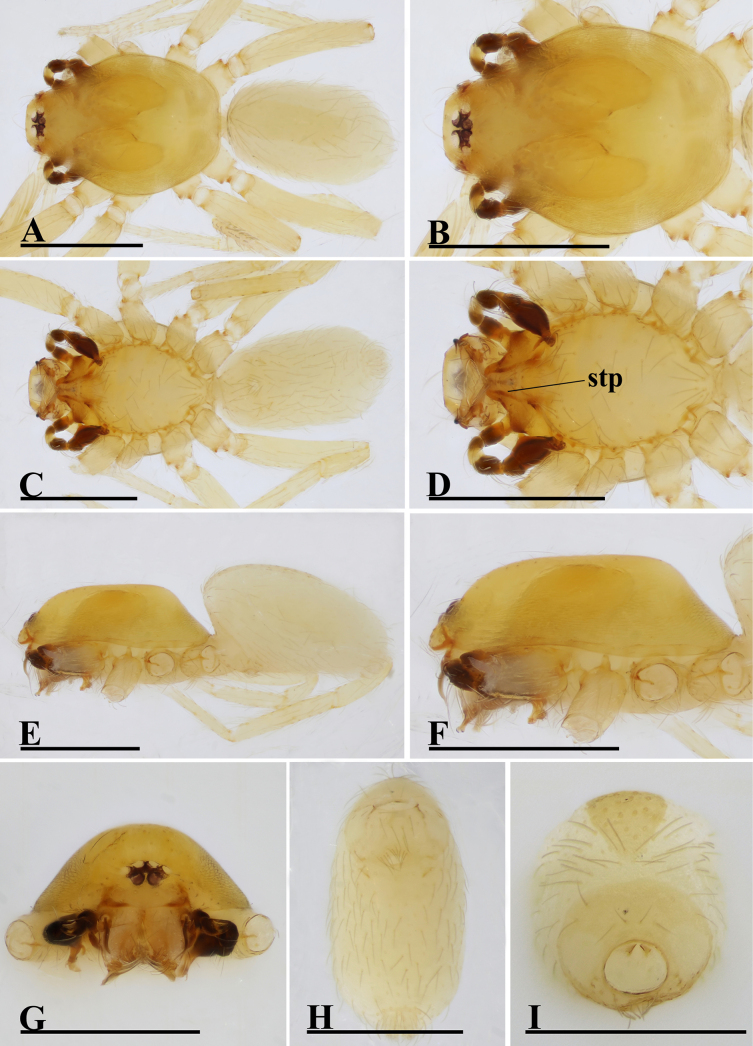
*Ischnothyreus luobidong* sp. nov., male holotype. **A**. Habitus, dorsal view; **B**. Cephalothorax, dorsal view; **C**. Habitus, ventral view; **D**. Cephalothorax, ventral view; **E**. Habitus, lateral view; **F**. Cephalothorax, lateral view; **G**. Cephalothorax, anterior view; **H**. Abdomen, ventral view; **I**. Abdomen, anterior view. Abbreviation: stp = strong, tooth-like projection. Scale bars: 0.4 mm.

**Figure 14.**
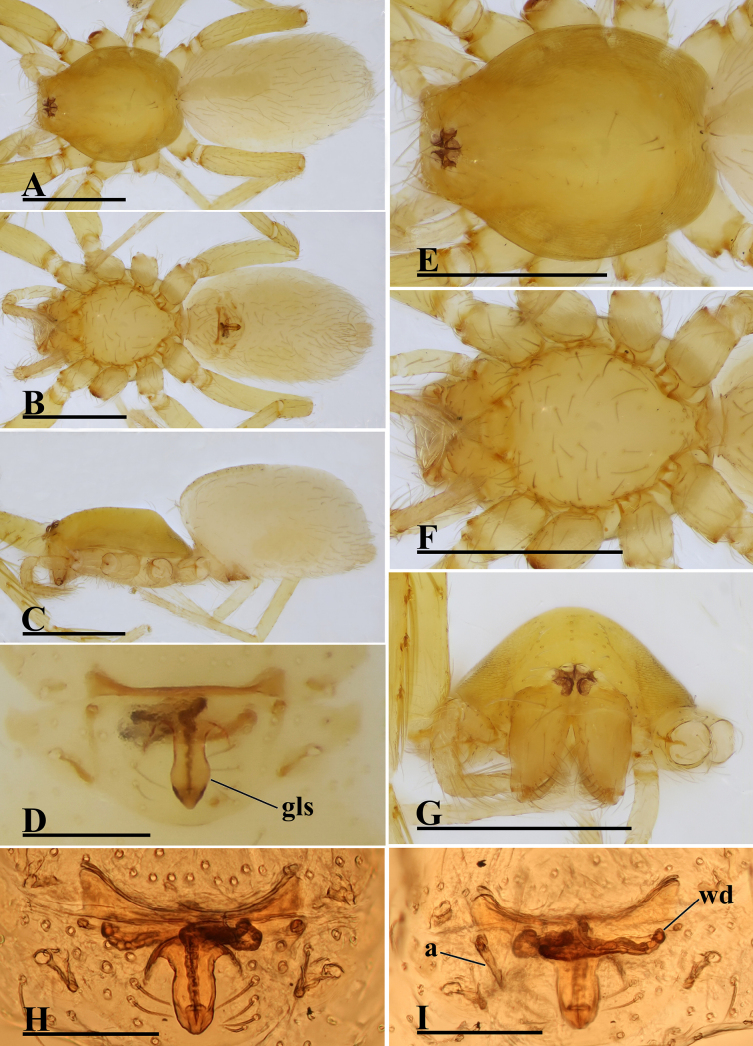
*Ischnothyreus luobidong* sp. nov., female paratype. **A**. Habitus, dorsal view; **B**. Habitus, ventral view; **C**. Habitus, lateral view; **D**. Epigastric region, ventral view; **E**. Cephalothorax, dorsal view; **F**. Cephalothorax, ventral view; **G**. Cephalothorax, anterior view; **H**. Endogyne, ventral view (cleared); **I**. Endogyne, dorsal view (cleared). Abbreviations: a = apodeme; gls = goblet-like structure; wd = winding duct. Scale bars: 0.4 mm (**A–C, E–G**); 0.1 mm (**D, H, I**).

Figs 13, 14, 16 G, 17G, 19G–I, 23A–D

##### Common name.

Luobidong Weak-spotted Spider (落笔洞弱斑蛛).

##### Type material.

***Holotype***: China • ♂ (SYNU-F-2515); Hainan, Sanya City, Jiyang Dist., Lizhigou Town, Luobi Cave; 18°19'54.30"N, 109°32'56.80"E, 46 ± 16 m elev.; 17.IV.2012; Chen leg. ***Paratypes***: China • 2♂2♀ (SYNU-2516–2519); same data as holotype • 2♂1♀ (SYNU-2520–2522); same data as holotype.

##### Etymology.

The specific name is a noun in apposition taken from the type locality.

##### Diagnosis.

The new species is similar to *I. matang* Kranz-Baltensperger, 2011 in the goblet-like structure of endogyne but can be distinguished by the very small eyes (vs well developed; cf. Figs 13B, 14Eand Kranz-Baltensperger 2011: figs 28C, 29A), the dorsal abdominal scutum less than 1/3 of abdomen width in males and 1/6 in females (vs approximately 1/2 in males and 1/4 in females; cf. Figs 13A, 14Aand Kranz-Baltensperger 2011: figs 28A, 29A) and the thorn-like process of male chelicerae (vs absent; cf. Figs 16G, 17Gand Kranz-Baltensperger 2011: fig. 28D).

##### Description.

**Male (holotype). *Body***: habitus as in Fig. 13A, C, E; body length 1.24. ***Carapace***: 0.65 long, 0.48 wide; light yellow, oval in dorsal view, with egg-shaped patches behind eyes, pars cephalica slightly elevated in lateral view, surface of elevated portion of pars cephalica smooth, sides finely reticulate (Fig. 13B, F). ***Clypeus***: straight in frontal view, ALE separated from edge of carapace by 1.8× of their diameter (Fig. 13G). ***Eyes***: six, reduced and very small, ALE largest, ALE circular, PME and PLE oval; posterior eye row procurved from above; ALE separated by less than their radius, PME–PLE touching, ALE–PLE touching (Fig. 13B, G). ***Sternum***: longer than wide, pale yellow, surface smooth, sparse setae (Fig. 13D). ***Mouthparts***: chelicerae, endites and labium yellow; chelicerae straight, anterior face with thorn-like process (tlp) on disk-like hair base (dhb), fang groove with a few small and one larger denticles (Figs 16G, 17G); anteromedian tip of endites with one strong, tooth-like projection (stp) (Fig. 13D). ***Abdomen***: 0.67 long, 0.35 wide; dorsal scutum well sclerotized, pale yellow, covering less than 1/3 of abdomen width and approximately 2/3 of abdomen length; epigastric scutum, pale yellow, fused to postgastric scutum; postgastric scutum covering 1/2 of abdomen length (Fig. 13H). ***Legs***: pale yellow, leg spines longer than segment width, spine formula: femora: I p0-1-1; II p0-0-1; tibiae: I, II p2-1-1; r2-1-1; metatarsi: I, II p1-1-0; r1-1-0. Legs III and IV spineless. ***Palp***: dark reddish brown, trochanter with ventral projection; bulb with two ventral protuberances, distal end of bulb stout, with ventral lobe (vl), large triangular dorsal membrane (dm) and retrolateral lobe (rl) (Figs 19G–I, 23A–D).

**Female (paratype, SYNU-2518)**. Same as male except as noted. ***Body***: habitus as in Fig. 14A–C; body length 1.33. ***Carapace***: 0.58 long, 0.46 wide (Fig. 14E). ***Mouthparts***: chelicerae and endites unmodified (Fig. 14F). ***Abdomen***: 0.82 long, 0.48 wide; dorsal scutum covering 1/6 of abdomen width and approximately 1/2 of abdomen length; epigastric scutum well sclerotized, pale yellow, not fused to postgastric scutum (Fig. 14A–D). ***Endogyne***: with goblet-like structure (gls); winding duct (wd) strongly convoluted; apodemes (a) present (Fig. 14H, I).

##### Distribution.

Known only from the type locality.

#### Ischnothyreus wuzhishan

Taxon classificationAnimaliaAraneaeOonopidae

Tong & Li sp. nov.

C86E3CA9-668A-549A-8670-A852714BF4C0

https://zoobank.org/A3B627E9-333F-4965-A4B1-17A6723CAC11

**Figure 15.**
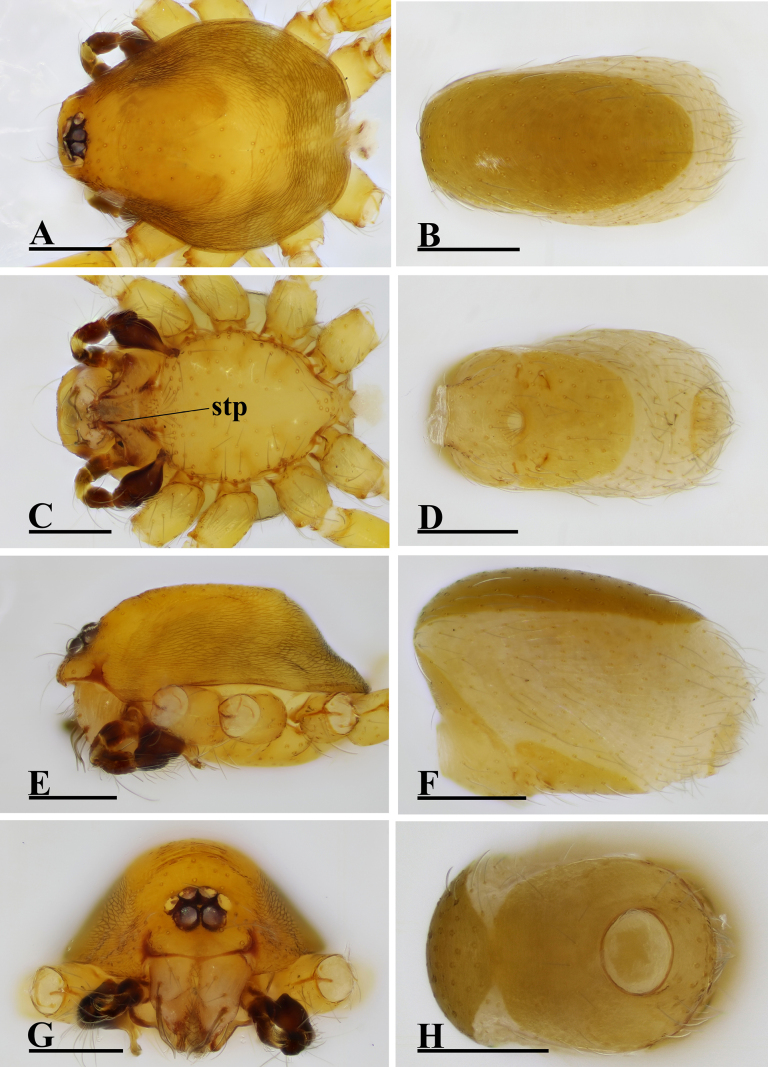
*Ischnothyreus wuzhishan* sp. nov., male holotype. **A**. Cephalothorax, dorsal view; **B**. Abdomen, dorsal view; **C**. Cephalothorax, ventral view; **D**. Abdomen, ventral view; **E**. Cephalothorax, lateral view; **F**. Abdomen, lateral view; **G**. Cephalothorax, anterior view; **H**. Abdomen, anterior view. Abbreviation: stp = strong, tooth-like projection. Scale bars: 0.2 mm.

**Figure 16.**
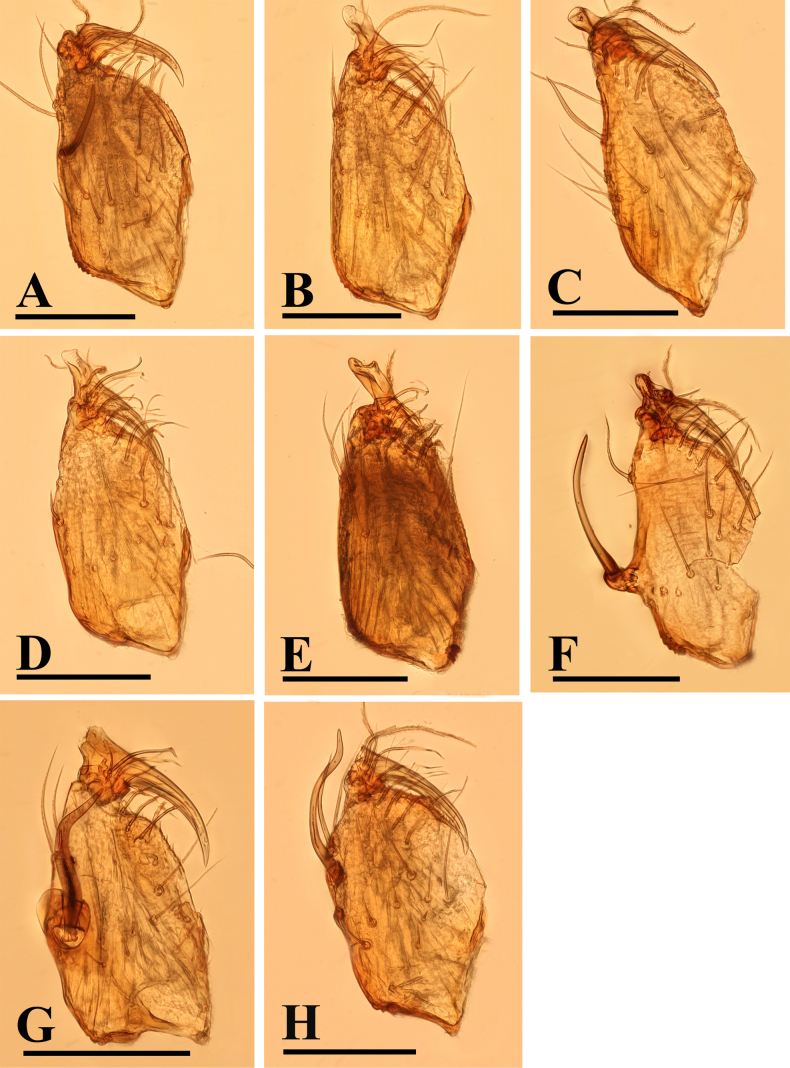
*Ischnothyreus* spp., male left chelicerae, anterior view. **A**. *I. bawangling* sp. nov.; **B**. *I. diaoluoshan* sp. nov.; **C**. *I. hongxin* sp. nov.; **D**. *I. jianfengling* sp. nov.; **E**. *I. limuling* sp. nov.; **F**. *I. liudao* sp. nov.; **G**. *I. luobidong* sp. nov.; **H**. *I. wuzhishan* sp. nov. Scale bars: 0.1 mm.

**Figure 17.**
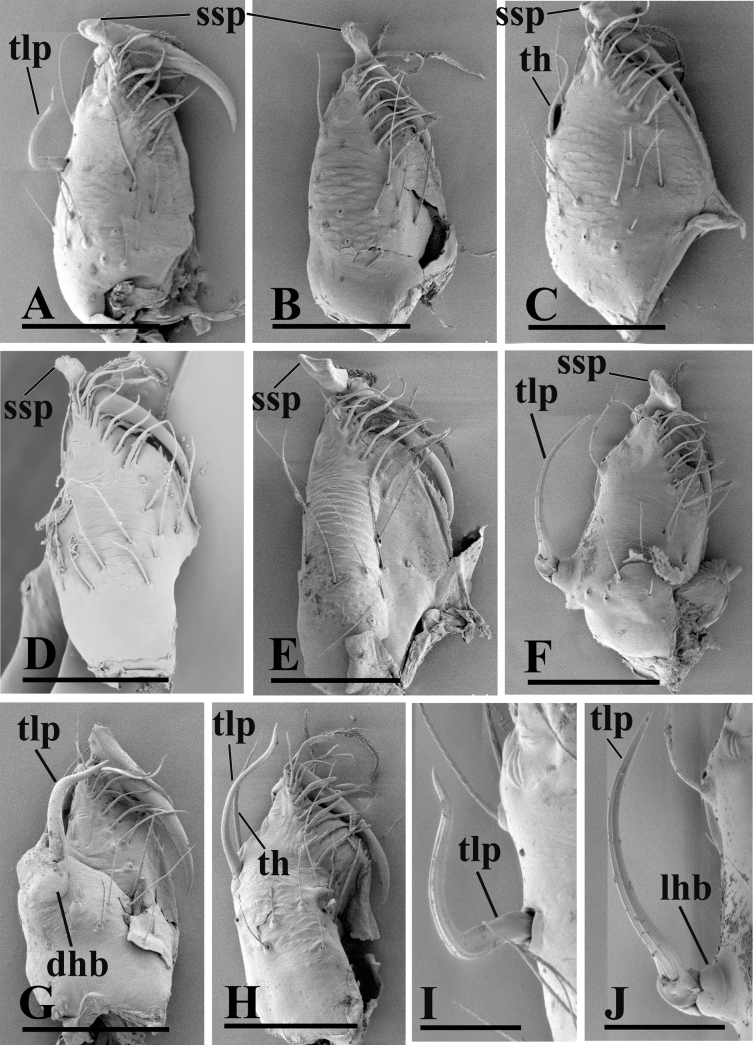
*Ischnothyreus* spp., male left chelicerae, anterior view, SEM. **A, I**. *I. bawangling* sp. nov.; **B**. *I. diaoluoshan* sp. nov.; **C**. *I. hongxin* sp. nov.; **D**. *I. jianfengling* sp. nov.; **E**. *I. limuling* sp. nov.; **F, J**. *I. liudao* sp. nov.; **G**. *I. luobidong* sp. nov.; **H**. *I. wuzhishan* sp. nov. Abbreviations: dhb = disk-like hair base; lhb = large hair base; ssp = slightly sclerotized process; th = thick hair; tlp = thorn-like process. Scale bars: 0.1 mm (**A–H**); 0.02 mm (**I, J**).

Figs 15, 16 H, 17H, 19J–L, 23E–H

##### Common name.

Wuzhishan Weak-spotted Spider (五指山弱斑蛛).

##### Type material.

***Holotype***: China • ♂ (SYNU-F-1709); Hainan, Wuzhishan City, Wuzhishan National Natural Reserve; 18°54'07.81"N, 109°41'14.10"E, 920 ± 24 m elev.; 2.IV.2012; Chen leg.

##### Etymology.

The specific name is a noun in apposition taken from the type locality.

##### Diagnosis.

The new species is similar to *I. microphthalmus* Richard, 2016 in the large dorsal abdominal scutum and very small eyes but can be distinguished by the reticulate carapace (vs smooth; cf. Fig. 15A and Richard et al. 2016: fig. 56A, F), the thorn-like process of male chelicerae (vs absent; cf. Figs 16H, 17Hand Richard et al. 2016: fig. 56C) and the large dorsal membrane (vs absent; cf. Figs 19J, 23Eand Richard et al. 2016: fig. 58A, C, E).

**Figure 18.**
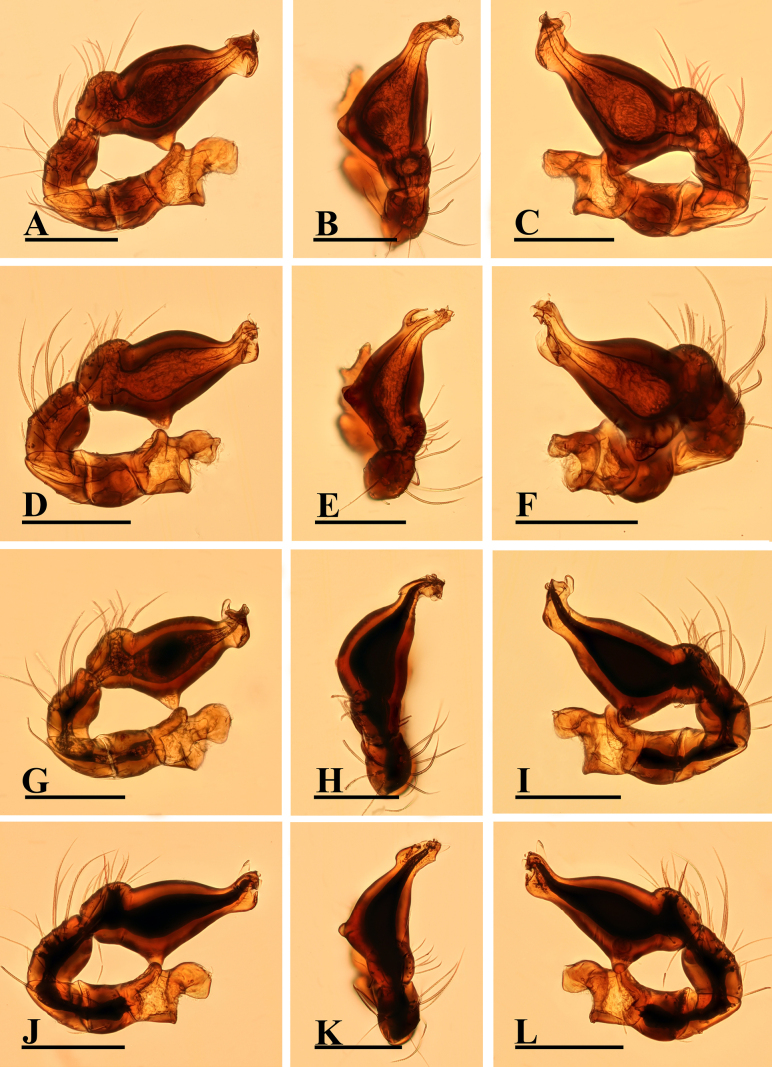
*Ischnothyreus* spp., male holotype, left palp. **A–C**. *I. bawangling* sp. nov.; **D–F**. *I. diaoluoshan* sp. nov.; **G–I**. *I. hongxin* sp. nov.; **J–L**. *I. jianfengling* sp. nov. **A, D, G, J**. Prolateral view; **B, E, H, K**. Dorsal view; **C, F, I, L**. Retrolateral view. Scale bars: 0.1 mm.

**Figure 19.**
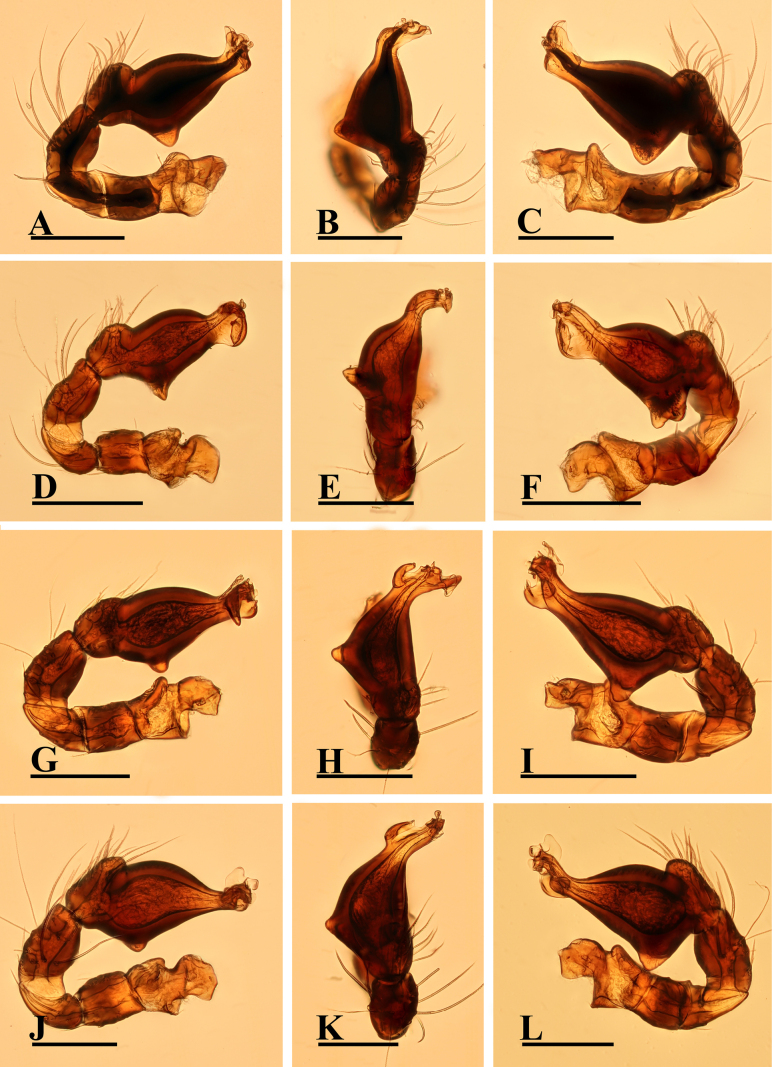
*Ischnothyreus* spp., male holotype, left palp. **A–C**. *I. limuling* sp. nov.; **D–F**. *I. liudao* sp. nov.; **G–I**. *I. luobidong* sp. nov.; **J–L**. *I. wuzhishan* sp. nov. **A, D, G, J**. Prolateral view; **B, E, H, K**. Dorsal view; **C, F, I, L**. Retrolateral view. Scale bars: 0.1 mm.

**Figure 20.**
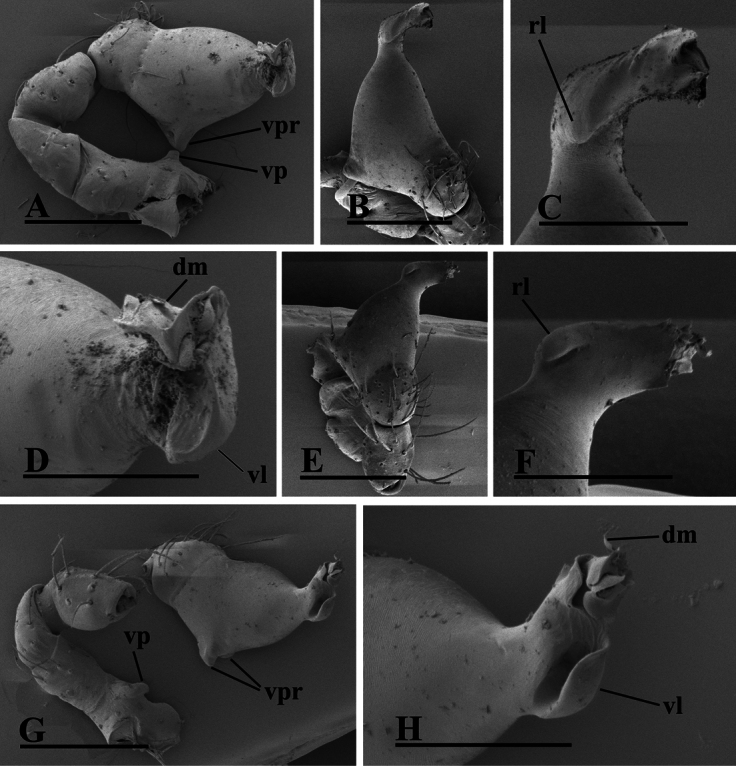
*Ischnothyreus bawangling* sp. nov., male holotype. **A–D**. SEM; *Ischnothyreus diaoluoshan* sp. nov., male holotype; **E–H**. SEM; **A, G**. Left palp, prolateral view; **B, E**. Left palp, dorsal view; **C, F**. Distal part of palpal bulb, dorsal view; **D, H**. Distal part of palpal bulb, prolateral view. Abbreviations: dm = dorsal membrane; rl = retrolateral lobe; vl = ventral lobe; vp = ventral projection; vpr = ventral protuberances. Scale bars: 0.1 mm (**A, B, E, G**); 0.05 mm (**C, D, F, H**).

**Figure 21.**
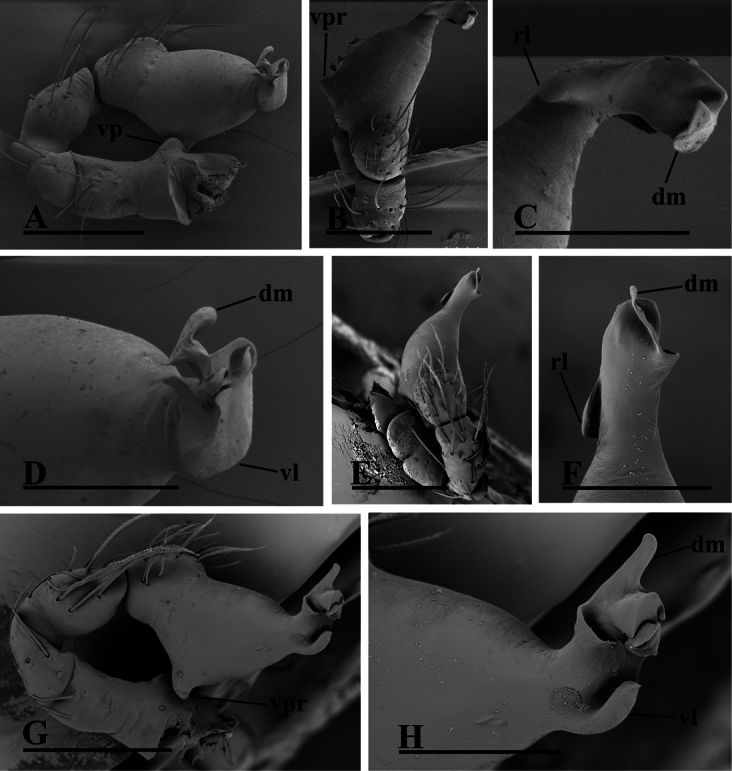
*Ischnothyreus hongxin* sp. nov., male holotype. **A–D**. SEM; *Ischnothyreus jianfengling* sp. nov., male holotype, **E–H**. SEM; **A, G**. Left palp, prolateral view; **B, E**. Left palp, dorsal view; **C, F**. Distal part of palpal bulb, dorsal view; **D, H**. Distal part of palpal bulb, prolateral view. Abbreviations: dm = dorsal membrane; rl = retrolateral lobe; vl = ventral lobe; vp = ventral projection; vpr = ventral protuberances. Scale bars: 0.1 mm (**A, B, E, G**); 0.05 mm (**C, D, F, H**).

**Figure 22.**
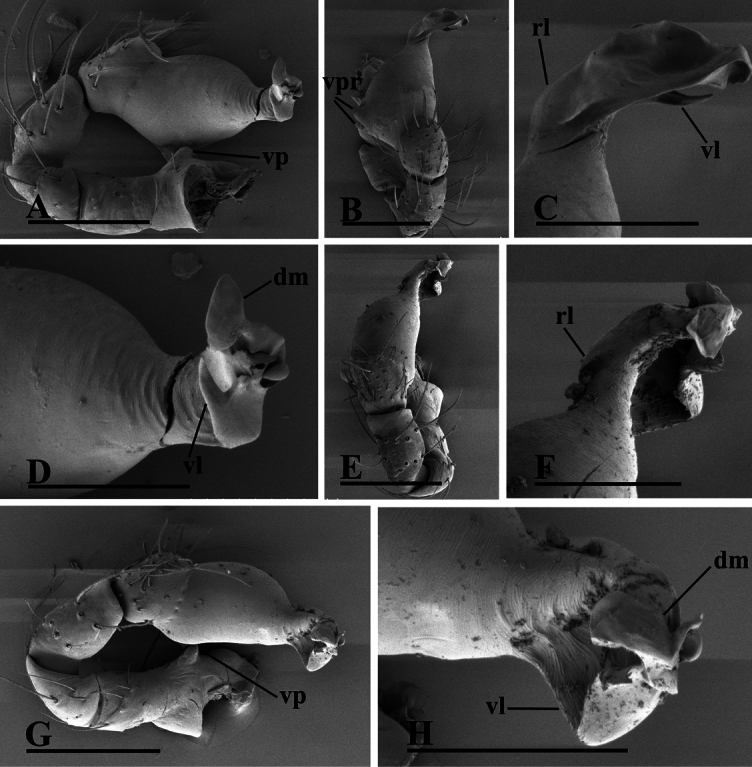
*Ischnothyreus limuling* sp. nov., male holotype. **A–D**. SEM; *Ischnothyreus liudao* sp. nov., male holotype; **E–H**. SEM; **A, G**. Left palp, prolateral view; **B, E**. Left palp, dorsal view; **C, F**. distal part of palpal bulb, dorsal view; **D, H**. Distal part of palpal bulb, prolateral view. Abbreviations: dm = dorsal membrane; rl = retrolateral lobe; vl = ventral lobe; vp = ventral projection; vpr = ventral protuberances. Scale bars: 0.1 mm (**A, B, E, G**); 0.05 mm (**C, D, F, H**).

**Figure 23.**
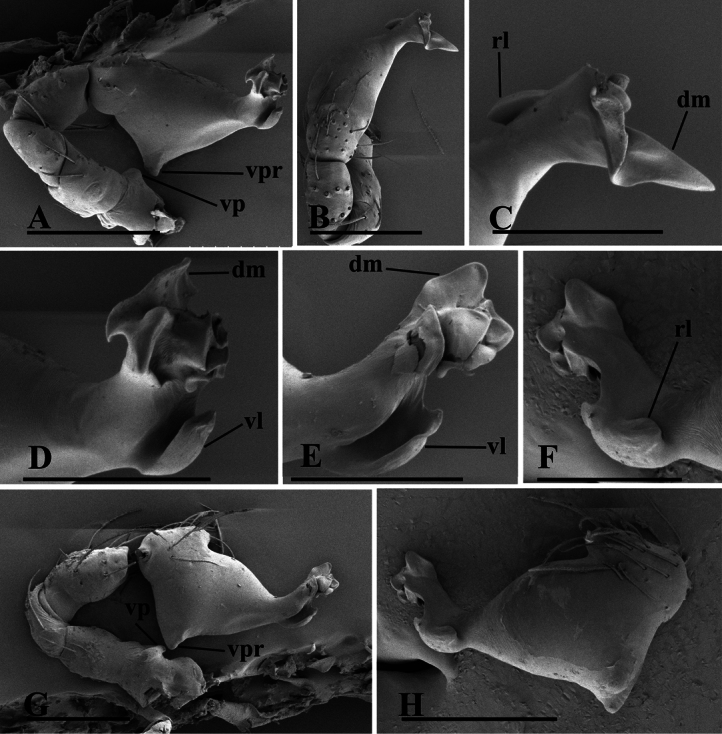
*Ischnothyreus luobidong* sp. nov., male holotype. **A–D**. SEM; *Ischnothyreus wuzhishan* sp. nov., male holotype, **E–H**. SEM. **A, G**. Left palp, prolateral view; **B**. Left palp, dorsal view; **C**. Distal part of palpal bulb, dorsal view; **D, E**. Distal part of palpal bulb, prolateral view; **F**. Distal part of palpal bulb, retrolateral view; **H**. Palpal bulb, retrolateral view. Abbreviations: dm = dorsal membrane; rl = retrolateral lobe; vl = ventral lobe; vp = ventral projection; vpr = ventral protuberances. Scale bars: 0.1 mm (**A, B, G, H**); 0.05 mm (**C–F**).

##### Description.

**Male (holotype). *Body***: body length 1.22. ***Carapace***: 0.71 long, 0.57 wide; yellowish brown, broadly oval in dorsal view, with egg-shaped patches behind eyes, pars cephalica slightly elevated in lateral view, surface of elevated portion of pars cephalica smooth, sides strongly reticulate (Fig. 15A, E). ***Clypeus***: straight in frontal view, ALE separated from edge of carapace by 1.2× of their diameter (Fig. 15G). ***Eyes***: six, reduced and very small, ALE largest, ALE circular, PLE oval, PME squared; posterior eye row procurved from above; ALE touching, PME–PLE touching, ALE–PLE touching (Fig. 15A, G). ***Sternum***: longer than wide, yellow, surface smooth, sparse setae (Fig. 15C). ***Mouthparts***: chelicerae, endites and labium yellow; chelicerae straight, anterior face with thorn-like process (tlp) and thick hair (th), fang groove with a few small denticles (Figs 16H, 17H); anteromedian tip of endites with one strong, tooth-like projection (stp) (Fig. 15C). ***Abdomen***: 0.62 long, 0.34 wide; dorsal scutum well sclerotized, yellowish brown, covering almost all of abdomen width and approximately 5/6 of abdomen length, fused to epigastric scutum (Fig. 15B, H); epigastric scutum, yellow, fused to postgastric scutum; postgastric scutum covering 2/3 of abdomen length (Fig. 15D). ***Legs***: yellow, leg spines longer than segment width, spine formula: femora: I p0-1-1; II p0-0-1; tibiae: I, II p2-1-1; r2-1-1; metatarsi: I, II p1-1-0; r1-1-0. Legs III and IV spineless. ***Palp***: dark reddish brown, trochanter with ventral projection; bulb with two ventral protuberances, distal end of bulb stout, with large ventral lobe (vl), dorsal membrane (dm) and retrolateral lobe (rl) (Figs 19J–L, 23E–H).

**Female**. Unknown.

##### Distribution.

Known only from the type locality.

## Supplementary Material

XML Treatment for Ischnothyreus bawangling

XML Treatment for Ischnothyreus diaoluoshan

XML Treatment for Ischnothyreus hongxin

XML Treatment for Ischnothyreus jianfengling

XML Treatment for Ischnothyreus limuling

XML Treatment for Ischnothyreus liudao

XML Treatment for Ischnothyreus luobidong

XML Treatment for Ischnothyreus wuzhishan

## Additional information

### Artificial Intelligence (AI) use

The authors accept full responsibility for the content of the manuscript, including the disclosure of any use of AI.

Regarding the use of AI in the preparation of this manuscript, the authors declare the following: The authors used Doubao for language refinement in the manuscript.

### Author ORCIDs

S. Shi https://orcid.org/0009-0003-6482-9926

D. Bian https://orcid.org/0000-0002-9324-8026

Y. Tong https://orcid.org/0000-0002-4348-7029

S. Li https://orcid.org/0000-0002-3290-5416

### Data availability

All of the data that support the findings of this study are available in the main text.

## References

[B1] Edward KL, Harvey MS (2014) Australian goblin spiders of the genus *Ischnothyreus* (Araneae, Oonopidae). Bulletin of the American Museum of Natural History 389: 1–144. 10.1206/865.1

[B2] Fu H, Wang Z, Sun Y, Tong Y, Bian D (2023) A new species of *Ischnothyreus* Simon, 1893 (Araneae, Oonopidae) from Guangdong Province, China. Biodiversity Data Journal 11: e105283. 10.3897/BDJ.11.e105283 PMC10257077 37305451

[B3] Huang Y, Tong Y, Bian D, Li S (2021) One new species of the genus *Ischnothyreus* Simon, 1893 and re-description of *I. yueluensis* Yin & Wang, 1984 from China (Araneae, Oonopidae). Biodiversity Data Journal 9: e66843. 10.3897/BDJ.9.e66843 PMC8134419 34025143

[B4] Khmelik VV, Kozub D, Glazunov A (2005) Helicon Focus 3.10.3. https://www.heliconsoft.com/heliconsoft-products/helicon-focus/ [accessed 10 May 2026]

[B5] Kranz-Baltensperger Y (2011) The oonopid spider genus *Ischnothyreus* in Borneo (Oonopidae, Araneae). Zootaxa 2939: 1–49. 10.11646/zootaxa.2939.1.1

[B6] Kranz-Baltensperger Y (2012) Three new species of the oonopid spider genus *Ischnothyreus* (Araneae: Oonopidae) from Tioman Island (Malaysia). Zootaxa 3161: 37–47. 10.11646/zootaxa.3161.1.3

[B7] Liu K, Henrard A, Xiao Y, Xu X (2019) On three new oonopid species from China and the discovery of the male *Orchestina bialata* Liu, Xiao & Xu, 2016 (Araneae: Oonopidae). Zootaxa 4701(3): 235–256. 10.11646/zootaxa.4701.3.2 32229940

[B8] Richard M, Graber W, Kropf C (2016) The goblin spider genus *Ischnothyreus* (Araneae, Oonopidae) in Java and Sumatra. Zootaxa 4151(1): 1–99. 10.11646/zootaxa.4151.1.1 27615819

[B9] Song C, Tong Y, Bian D, Zhang Z (2024) Two new species and one new record of *Ischnothyreus* Simon, 1893 (Araneae, Oonopidae) from China. Biodiversity Data Journal 12: e122100. 10.3897/BDJ.12.e122100 PMC11026908 38645471

[B10] Tong Y, Li S (2008) The oonopid spiders (Araneae: Oonopidae) from Hainan Island, China. Raffles Bulletin of Zoology 56: 55–66.

[B11] Tong Y, Li S (2012) Four new species of the genus *Ischnothyreus* from Hainan Island, China (Araneae, Oonopidae). Zootaxa 3352: 25–39. 10.11646/zootaxa.3352.1.3 26120729

[B12] Tong Y, Li S (2013) Six new species of oonopid spiders from Champasak, Laos (Araneae, Oonopidae). Zootaxa 3709: 71–88. 10.11646/zootaxa.3709.1.3 26240897

[B13] Tong Y, Li S (2014) A survey of oonopid spiders in Taiwan with descriptions of three new species. ZooKeys 396: 67–86. 10.3897/zookeys.396.7033 PMC3978266 24715794

[B14] Tong Y, Bian D, Li S (2023) Three new species of the genus *Ischnothyreus* Simon, 1893 and the discovery of the male of *I. linzhiensis* Hu, 2001 from Tibet, China (Araneae, Oonopidae). ZooKeys 1152: 119–131. 10.3897/zookeys.1152.100341 PMC10193446 37214739

[B15] Tong Y, He J, Li S (2018) A new species of the genus *Ischnothyreus* Simon, 1893 from Chongqing, China (Araneae, Oonopidae). Journal of Shenyang Normal University (Natural Science edition) 36(1): 10–15.

[B16] Tong Y, Koh JKH, Tong X, Li S (2016) Five new species of the genus *Ischnothyreus* Simon, 1893 from Singapore. ZooKeys 618: 39–66. 10.3897/zookeys.618.9451 PMC5102049 27853399

[B17] Tong Y, Li S, Bian D (2020) Taxonomic study of the genus *Ischnothyreus* Simon, 1893 from Myanmar (Araneae, Oonopidae). ZooKeys 993: 1–26. 10.3897/zookeys.993.57676 PMC7700760 33304114

[B18] Tong Y, Sun X, Li S, Bian D (2021) Taxonomic study of the genus *Ischnothyreus* (Araneae, Oonopidae) from Xishuangbanna Rainforest, southwestern China. ZooKeys 1034: 165–197. 10.3897/zookeys.1034.63388 PMC8093188 33958929

[B19] WSC (2026) World Spider Catalog. Version 27. Natural History Museum Bern. 10.24436/2 [accessed on 4 July 2026]

